# Ligand-free reductive amination *via* Pd-coated mechanocatalysis

**DOI:** 10.1039/d5cc04707b

**Published:** 2025-10-23

**Authors:** Maximilian Wohlgemuth, Sarah Schmidt, Lars Beißel, Lars Borchardt

**Affiliations:** a Inorganic Chemistry I, Ruhr University Bochum Universitätsstraße 150 44801 Bochum Germany Maximilian.wohlgemuth@rub.de Lars.borchardt@rub.de

## Abstract

Reductive amination under direct mechanocatalysis was enabled by a sequential two-frequency milling protocol, separating imine formation and hydrogenation. Strongly hydrophobic liquid additives accelerated the reaction by enhancing water exclusion. This approach allowed amine yields of up to 88% and was applicable to various aldehydes, ketones, and primary or secondary amines.

## Abstract 2

Reductive amination under direct mechanocatalysis was enabled by a sequential two-frequency milling protocol, separating imine formation and hydrogenation.

## Graphical abstract

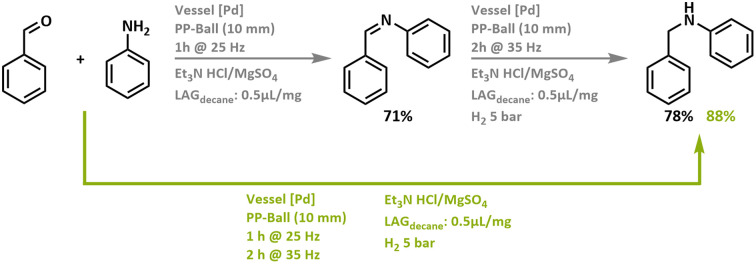

Reductive amination is a central C–N bond-forming reaction in organic synthesis, widely employed for the preparation of active pharmaceutical ingredients, agrochemicals, and fine chemicals.^1–3^ Classical protocols typically rely on solution-phase chemistry using stoichiometric reducing agents such as borohydrides^4–7^ or hydrosilanes,^8–10^ often generating toxic side products. As a sustainable alternative, molecular hydrogen (H_2_) has been employed in combination with transition metal catalysts to achieve reductive amination with improved sustainability.^11–13^ However, such protocols typically rely on solvent-based reaction media and often require elevated temperatures, pressures, or complex ligand systems. The application of H_2_-driven reductive amination under solvent-free conditions remains notably underexplored. Mechanochemistry has emerged as an attractive strategy to circumvent the limitations of conventional synthesis, enabling efficient, solvent-free transformations by mechanical activation. In this context, direct mechanocatalysis, wherein catalytically active metals are integrated directly into the milling vessel or balls, offers a suitable approach to conduct catalytic reactions without external ligands, solvents, or powdered catalysts.^14–19^ Recent advances have demonstrated hydrogenation of C=C, C=O bonds and the reduction of nitro-groups *via* direct mechanocatalysis using gaseous H_2_ under ambient conditions.^20^ However, the application of this strategy to reductive amination—a more complex, multicomponent reaction—remains unexplored. Herein, we report the first example of a reductive amination using molecular hydrogen under direct mechanocatalytic conditions. This approach employs Pd-coated milling vessels and polypropylene balls under ambient hydrogen pressure, enabling the reaction with primary and secondary amines as well as their corresponding hydrochloride salts. We identified that it is beneficial to perform the reaction steps (condensation and hydrogenation) under two different milling conditions to suppress the formation of by-products. The advantage of mechanochemical conversion here is that the mill can change the parameters without pausing or further intervention. Notably, the catalyst coatings were stable over multiple cycles, allowing catalyst reuse with minimal leaching, which is important for use in the fine chemical industry.

The reductive amination of 1 mmol benzaldehyde with 2 eq. aniline was used as a model reaction using a palladium plated 14 mL gasable milling vessel and a 10 mm polypropylene milling ball with 1 g of magnesium sulfate (0.5 g) and triethylamine hydrochloride (0.5 g) at 5 bar H_2_ pressure for two hours at 30 Hz. With these reaction conditions, 12% of the amine product and 41% of the imine were formed. As side products, benzylalcohol and *N*,*N*-dibenzylaniline were observed. The yields of the amine product and the side products were quantified using HPLC-analysis, while the imine intermediate was quantified with NMR-spectroscopy using cyclooctane as an internal standard. The effect of hydrogen pressure and catalyst coating was examined first. The change of the hydrogen pressure to one bar results in just little changes in the obtained products (Table 1, entry 2) while without the palladium coating no hydrogenation reactions were observed (Table 1, entry 1). To minimize abrasion of the palladium-coated milling vessel (<1 ppm; approx. 1.1 μg, measured by ICP-OES) and to provide a soft bulk material, we initially employed a mixture of magnesium sulfate and triethylamine hydrochloride. Beyond protecting the catalytic surface, this combination also gave the best performance for amine formation, which can partly be ascribed to the water-withdrawing character of magnesium sulfate that promotes imine formation. In comparison, potassium carbonate reduced the amount of alcohol side product (4%) but also lowered the amine yield (5%). With acidic bulk materials such as potassium hydrogen sulfate, the alcohol became the predominant product (Table 1, entry 5), which can be attributed to the acidic character that promotes the hydrogenation of the carbonyl double bond. In the next steps we investigated the energy influence by changing the milling frequencies. We found that a high milling frequency (30, 35 Hz) leads to high amounts of alcohol formation (10 and 23%) while lower frequencies (20, 25 Hz) did not form any amine product after 2 h of reaction time. However, the formation of the imine has its peak of 78% at a milling frequency of 25 Hz (Table 1A, entry 6). We found out that a high milling frequency (30, 35 Hz) leads to high amounts of alcohol formation (10 and 23%) while lower frequencies (20, 25 Hz) did not form any amine product after 2 h of reaction time. This suggests that the initial condensation step proceeds even at low frequencies, whereas the subsequent hydrogenation requires higher energy input. If the milling energy is too high from the beginning, the aldehyde is simultaneously hydrogenated, resulting in the undesired alcohol by-product, while at 25 Hz the energy is simply insufficient to promote hydrogenation. Based on these observations, we decided to split our reaction into two phases, one with low energy input (25 Hz) and one with high energy input (35 Hz), which can be preprogrammed in the milling device. From this, we divided the following reactions into two different cycles and could no longer find any alcohol as a byproduct (neither in the GC nor in the NMR). First, we let the reaction mill at 25 Hz for 1 h and then increased the frequency to 35 Hz for one hour, without having to adjust anything on the mill in between. With this reaction we were able to obtain an amine yield of 24% after 2 h reaction time. By increasing the reaction time of the second step we were able to increase the yield of the amine to 71% in a total reaction time of 3 h (1 h at 25 Hz, 2 h at 35 Hz). At a higher reaction time at 35 Hz the “over alkylation” of the amine was observed (see SI, Fig. S4). Mechanochemical reactions are usually carried out under solvent-free conditions, and the resulting mixtures are often powdery, which can lead to clumping or snowballing of the powder on the ball, which significantly affects mixing. To adjust the rheology and enhance the mixing efficiency, small amounts of liquid additives—commonly referred to as liquid-assisted grinding (LAG)—were introduced. These liquids do not participate in the reaction but can promote homogenization. A range of liquid additives with varying polarity were investigated (see SI, Fig. S6). Polar or basic solvents such as acetonitrile and triethylamine resulted in reduced conversions, with product yields below 50% (see SI, Fig. S6). In contrast, the use of non-polar, non-aromatic solvents such as decane and cyclohexane led to a notable increase in the amine yield, reaching 71% (Table 1B entry 5) and 69%, respectively (see SI, Fig. S5). The improved yields observed with non-polar additives such as *n*-decane are likely due to two synergistic effects: enhanced hydrogen solubility and more favorable solubility behavior for key intermediates. Although toluene also dissolves hydrogen well, it afforded significantly lower yields (32%, Table 1, entry 13). This discrepancy can be attributed to the following: (1) *n*-decane dissolves both the imine intermediate and the amine product more efficiently; (2) toluene appears less effective in solubilizing the imine; and (3) the higher hydrophobicity of *n*-decane reduces water retention in the reaction zone. The latter effect likely shifts the condensation equilibrium toward imine formation by suppressing hydrolysis, thereby enhancing the overall conversion in this equilibrium-limited system. Using a slight excess of the amine substrate increased the overall product yield to as high as 88%. This improvement arises from the equilibrium nature of the initial condensation between the carbonyl compound and the amine. A higher amine concentration drives the equilibrium toward imine formation, thereby generating more of the reactive intermediate for subsequent hydrogenation. In addition, the amine excess effectively suppressed over-alkylation, preventing the formation of tertiary amines. In addition, we performed the reaction without palladium and hydrogen using NaBH_4_ to benchmark our system against established approaches. The NaBH_4_ system afforded ∼90% yield of the amine, albeit with ∼10% alcohol as a side product (see SI, chapter 1.7). In comparison, our method provides only slightly lower yields (<5% difference) but proceeds without side products and without the need for toxic reducing agents, thus representing a safer and more sustainable system. The sustainability of our system is highlighted by the fact that the catalyst (milling vessel) can be reused over five consecutive runs with constant yields of ∼88%, without any significant loss of activity (see SI, Chapter 1.8).

Optimization of the reaction conditions for the reductive amination with benzaldehyde and aniline *via* direct mechanocatalysis. A: reactions were done with the one milling frequency. B: reactions were done with two different milling frequencies without any purification or further intervention

**Table:** 

A	One milling step			
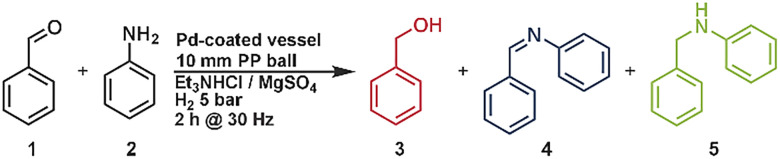				
Entry	Variation from standard conditions	Yield of 3 (%)	Yield of 4 (%)	Yield of 5 (%)
1	None	10	41	12
2	No Pd-coating	—	73	—
3	1 bar H_2_ pressure	7	42	9
4	K_2_CO_3_ as bulk	4	18	5
5	KHSO_4_ as bulk	57	12	—
6	25 Hz	—	78	4
7	35 Hz	23	48	16

a The adjusted reaction conditions were adopted from entry 6 with the shown changes.

**Table:** 

B	Two milling steps			
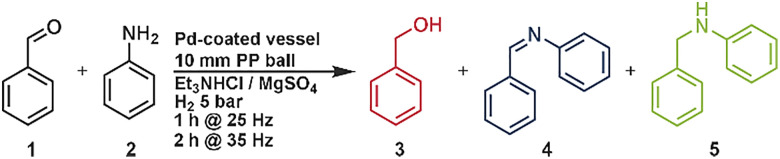				
Entry	Variation from standard conditions	Yield of 3 (%)	Yield of 4 (%)	Yield of 5 (%)
1	None	—	11	65
2	1 h at 25 Hz, 1 h at 35 Hz	—	42	24
3	1 h at 25 Hz, 3 h at 35 Hz	—	8	61
4	Liquid additive of 0.2 μL mg^−1^ decane	—	14	54
5	Liquid additive of 0.5 μL mg^−1^ decane	—	11	71
6	Liquid additive of 0.5 μL mg^−1^ toluene	—	20	32
7	2 eq. benzaldehyde^a^	10	27	31
**8**	**3 eq. aniline** a	**—**	**2**	**88**
9	4 eq. aniline^a^	—	3	74

Benzylic aldehydes, including benzaldehyde, exhibited the highest reactivity under the developed mechanocatalytic conditions, consistently affording amine products in yields exceeding 70% (Fig. 1A). In contrast, aliphatic aldehydes were less reactive, with 13% for undecanol and 43% for phenylacetaldehyde. The observed trend is attributed to the increased electrophilicity of the carbonyl carbon in benzylic substrates, which facilitates nucleophilic attack by the amine. Ketones proved less reactive in reductive amination, with yields ranging from 31% for aliphatic ketones to 24% for benzylic analogs, highlighting the influence of steric and electronic effects. Short-chain aliphatic amines often pose practical challenges due to their volatility and toxicity. To circumvent this, amines were employed as their corresponding hydrochloride salts under mechanochemical conditions. Notably, methylamine hydrochloride and ethylamine hydrochloride delivered the desired products in around 70% yield, respectively, without requiring gaseous reagents or solvent-based dosing. Furthermore, aniline hydrochloride was found to perform comparably to its free base, making the process operationally simple and efficient. Secondary amines showed the same trend as aldehydes; here, the yields vary between 12 to 20%.

**Fig. 1:**
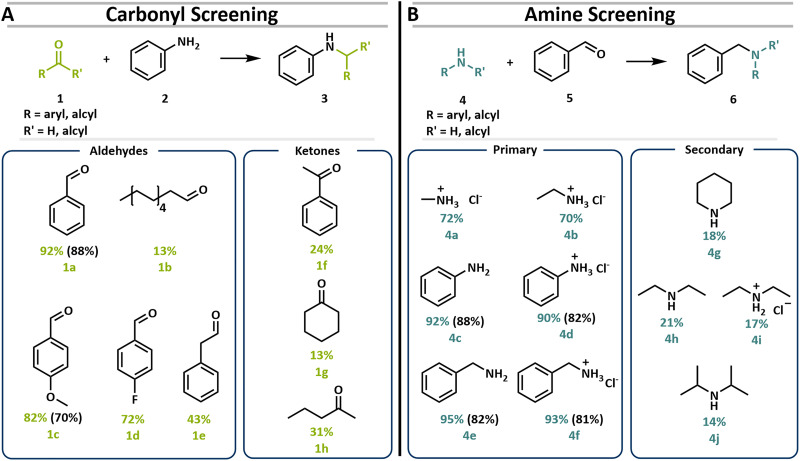
Substrate screening of different carbonyls with aniline (A) and benzaldehyde with different amines (B). Yield determined with ^1^H-NMR using cyclooctane as an internal standard in green and purified yield in black. For the substrate screening the amount of the imine intermediate as well as a potential over reacted tertiary amine were not investigated. The reaction was done with 1 mmol of aldehyde substrate and 4 eq. of the amine substrate were mixed with 1 g of a 1 : 1 mixture of triethylamine hydrochloride and magnesium sulfate with 5 bar hydrogen pressure for 1 h at 25 Hz and 2 h of 35 Hz in a MM500 mixer mill.

We report on the successful application of direct mechanocatalytic reductive amination using molecular hydrogen as the reducing agent. We demonstrate for the first time that varying the milling frequency within a single reaction cycle enables selective control over the two mechanistically distinct steps of imine formation and hydrogenation. By preprogramming sequential milling conditions, the transformation proceeds as a true one-pot and one-step protocol, avoiding intermediate handling and minimizing side product formation. In addition, we show that amines can be used in the form of their hydrochloride salts, which are solid, stable, and easy to handle. By this approach, even volatile and challenging-to-dose amines can be employed under solvent-free conditions, emphasizing the method's practical simplicity. These findings establish a robust and scalable strategy for sustainable amine synthesis and highlight the potential of direct mechanocatalysis.

We gratefully acknowledge the funding from the European Research Council (ERC) under the European Union's Horizon 2020 research and innovation program (“Mechanocat”, grant agreement no. 948521).

## Conflicts of interest

There are no conflicts to declare.

## Supplementary Material

CC-061-D5CC04707B-s001

## Data Availability

The data supporting this article have been included as part of the supplementary information (SI). Supplementary information is available. See DOI: https://doi.org/10.1039/d5cc04707b.
