# Supplementary material for: Ligand-free reductive amination via Pd-coated mechanocatalysis
Source: Chem Commun (Camb). 2025 Oct 23;61(95):18838–41. doi: 10.1039/d5cc04707b (PMC12581175; doi:10.1039/d5cc04707b)
Supplement: CC-061-D5CC04707B-s001 [file CC-061-D5CC04707B-s001.pdf]

Supplementary Information (SI) for ChemComm.
This journal is © The Royal Society of Chemistry 2025

Supporting Informations

Ligand-Free Reductive Amination via Pd-Coated Mechanocatalysis

Maximilian Wohlgemuth,*[a] Sarah Schmidt, [a] Lars Beißel [a] and Lars Borchardt*[a]

[a] Dr. M. Wohlgemuth, S. Schmidt, L. Beißel and Prof. Dr. L. Borchardt
Inorganic Chemistry I,
Ruhr-University Bochum
Universitätsstraße 150,
44801 Bochum (Germany)
E-mail: lars.borchardt@rub.de; Maximilian.wohlgemuth@rub.de

Table of contents

1. Experimental Section 3
   - 1.1 Used equipment and characterization techniques 3
   - 1.2 Preparation of the Catalyst 4
   - 1.3 Set-up 4
   - 1.4 General optimized reaction conditions 4
   - 1.5 Control experiments 5
   - 1.6 ICP-OES measurements 5
   - 1.7 Influence of Reducing Agents 5
   - 1.8 Catalyst cycling 6
   - 1.9 Screening of different milling parameter 8
2. Characterization 11
   - 2.1. Products 11
   - 2.2 Mass Spectra 15
   - 2.3 NMR 20

1. Experimental Section

1.1 Used equipment and characterization techniques

General. All reagents were obtained from commercial suppliers at least in synthesis grade purity and were used without further purification. Organic solvents used for LAG were obtained in analysis grade. The milling was carried out exclusively in a Retsch MM-500 vario ball mill. The milling vessels were custom made (see Fig. S1) from Steel (1.3505). The raw material for the vessels was obtained from MARKS GmbH in Eisenberg, Germany.

Gas chromatography-mass spectrometry (GCMS) was performed on a Shimadzu NEXIS 2030 gas chromatograph with a 30 m 0.25 mm ID, 5 % Diphenyl / 95 % dimethyl polysiloxane column. Electron ionization with an injection temperature of 200 °C was used. 1 mg of crude product was dissolved in 1 mL of DCM, filtered through a cotton filter and transferred to a GC vial.

High performance liquid chromatography (HPLC) was conducted on a Shimadzu Nexera LC-40 lite. A Nucleodur C18, 3 µm Reversed phase column from Machery-Nagel was used as stationary phase. A low-pressure gradient was used with a starting solvent mixture consisting of 80% water and 20% acetonitrile at a flowrate of 1 mL/min. At 2 min a solvent mixture of 50% of both solvents was reached going up to 80% acetonitrile and 20% water at a time from 3 min. Afterwards the solvent mixture was changed to 40% water and 60% acetonitrile was reached at 4.5 min. The measurement ended with a solvent mixture of 80% water and 20% acetonitrile at 5 min. For each measurement 3-5 mg of product mixture were dissolved in 3 mL of acetonitrile/water mixture (65:35), filtered through a syringe filter and transferred to a HPLC vial.

X-ray photoelectron spectroscopy (XPS). XPS Spectra were recorded on a Nexsa G2 Surface Analysis System. X-ray source type: Monochromated, micro-focused, high-efficiency Al Kα X-ray source. The spectra were obtained by scanning 20 times with 50 keV. Analyzer type: 180°, double-focusing, hemispherical analyzer with a 128-channel detector.

Inductively coupled plasma atomic emission spectroscopy (ICP-OES). ICP-OES measurements were performed on an Analytikjena Plasma Quant PQ 9000. Samples were taken from the purified product mixture, dissolved in an acetic acid acidified mixture of acetonitrile and water (60:40).

X-ray fluorescence spectroscopy (XRF). The XRF-spectra were obtained by a Vanta™Handheld XRF Analyzer with a 40 keV Rhodium X-ray tube.

Milling balls. The polymer milling balls were obtained from IHSD-Klarmann from Bamberg, Germany. Zirconia milling balls were obtained from RETSCH GmbH from Haan, Germany.

1.2 Preparation of the Catalyst

A pen plating system from Jentner was used to electroplate the milling vessels. After cleaning the vessels to remove grease and dust, a copper layer was applied as a ductile linker layer using a cyanide copper electrolyte of 35 g/L at 3 V. A nickel layer was added as a diffusion barrier using a 50 g/L nickel electrolyte at 3 V. For nickel catalysis, the coating process was stopped at this point. For rhodium- and palladium-catalysis, the coating process was continued by depositing gold using a 5 g/L electrolyte at 5 V to allow for a stable coating of the actual catalyst. For palladium catalysis, the layers were finalized with a 2 g/L electrolyte used at 3 V.

1.3 Set-up

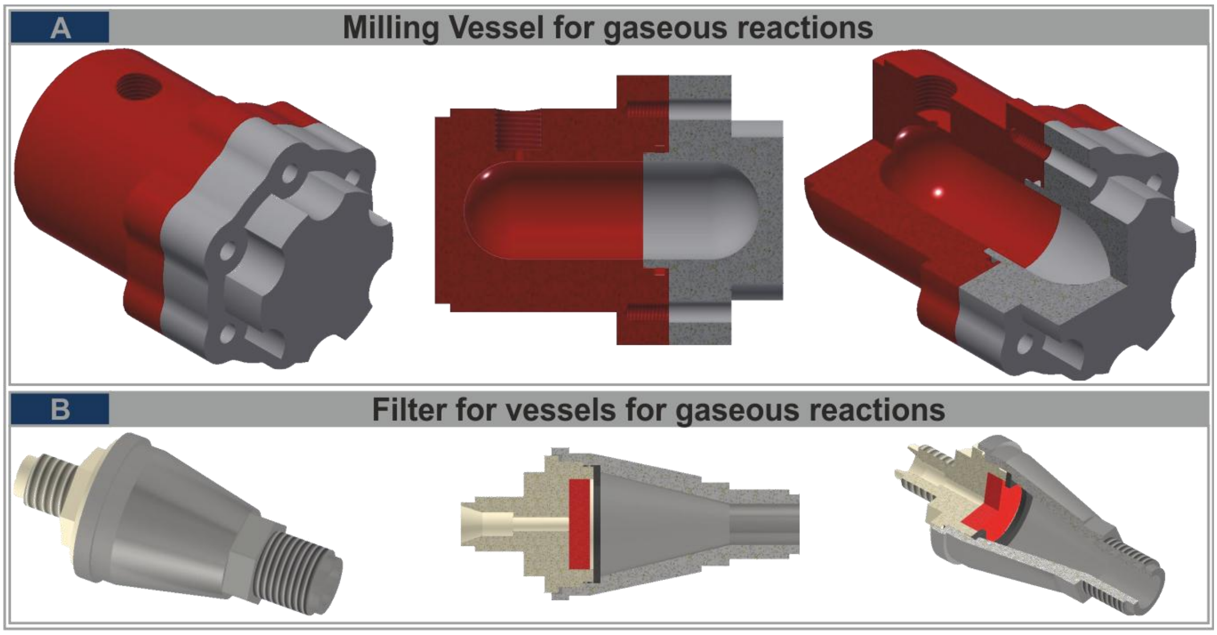

Figure S1: Design of the milling vessel used for the hydrogenation, allowing for gas supply during milling.

1.4 General optimized reaction conditions

1 mmol of benzaldehyde and 4 mmol anilin were employed as the substrates. A typical reaction used a 10 mm polypropylene ball, 1 g potassium carbonate and 1 mmol of substrate with η = 0.5 µL/mg decane as LAG in a 19 mL palladium-coated steel vessel. The vessel was flushed three times with 2 bar nitrogen to remove any oxygen from the set-up and then pressurized with 5 bar hydrogen. The reaction mixture was milled in a mixer mill MM500 by Retsch at a frequency of 25 Hz for 1 h and after this at 35 Hz for 2h. During the reaction, the vessel stayed connected to a 150 mL reservoir storing hydrogen at 5 bar to allow for resupply of any hydrogen used up in the reaction. The obtained crude product was dissolved into a system of ethyl acetate and water (ph value adjusted to 8). The organic phase was separated, dried with magnesium sulfate and the solvent removed on a rotavap. The product was obtained in the form of yellow to brown high viscous paste. For yield determination via quantitative 1H-NMR, the complete product was dissolved in deuterated chloroform. 1 mmol of dibromomethane was added as internal standard and the sample transferred to an NMR tube.

1.5 Control experiments

To proof if hydrogen and palladium are needed the reaction was performed under the in chapter 1.4 descripted conditions. The reaction without hydrogen 71% of the imine and traces (<2%) product were observed. Without palladium, 73% of the imine was observed without any amine product. To check if the reaction could work without mechanochemical energy, a reaction mixture was prepared on descripted in chapter 1.4 and the mixture was mixed for 15 min at 35 Hz, to generate a homogenized mixture. After this the closed milling vessel was transferred to a drying oven (80°C) and stored for 24 h. After this the crude mixture was purified and 21% of imine and 5% of the amine product was observed.

1.6 ICP-OES measurements

Since abrasion plays a crucial role in mechanochemistry, especially in direct mechanocatalysis. We prepared a different sample described on the following table. All samples were prepared with one milling ball and the reaction times of 1 h at 25 Hz followed by 2 h at 35 Hz with 5 bar hydrogen pressure, additionally substrates (1 mmol benzaldehyde and 4 mmol anilin) with 1 g of bulk material were added to the vessel.

Table S1: ICP-OES measurments of the crude mixture after a reaction under optimized conditions (descripted in 1.4) with different bulk conditions and milling times.[a]: reaction was performed with cleaned and ground eggshell waste.

Sample	Measured amount of Pd in the sample
Bulk: K_2SO_4	3.8 ppm
Bulk: $MgSO_4$	4.1 ppm
Bulk: $CaCO_3$[a]	2.1 ppm
4 h at 35 Hz	0.7 ppm

1.7 Influence of Reducing Agents

The performance of sodium borohydride ($NaBH_4$) as a reducing agent was compared to that of hydrogen gas (H_2) in a direct mechanocatalyzed reductive amination process (Table below). $NaBH_4$ afforded slightly higher yields of the target amine than H_2, likely due to its stronger reducing power, which enables efficient reduction of imine intermediates, resulting in faster conversion. However, the use of $NaBH_4$ also led to the formation of alcohol by-products. In contrast, although H_2 provided lower yields, it still promoted reductive amination and is gaining interest as a green, sustainable alternative that produces minimal by-products.

Table S2: Influence of different reducing agents. Reaction conditions: 1 mmol benzaldehyde, 4 mmol aniline, 1 g of bulk material (50:50 magnesium sulfate:Triethylamine hydrochloride) were milled with one 10 mm polypropylene ball in a 19 mL palladium-coated steel gas vessel for 2 h in a MM500 at 5 bar H_2, LAG: Decane with η=0.5 µL/mg.

Reducing agent	Amine yield / %	Imine yield / %	Alcohol yield / %
$NaBH_4$	91	0	8
H_2	88	2	0
None	Traces	71	0

1.8 Catalyst cycling

The optimized reaction conditions (see 1.4) were applied in a series of consecutive reactions using the same Pd-coated milling vessel without renewing the palladium layer. Only the amine yield is reported, as it directly reflects the activity of the palladium coating. The results of these experiments are summarized in Table S3.

Table S3: Catalyst cycling.

Reaction	Amine Yield / %
1	88.4
2	87.7
3	88.3
4	88.6
5	86.9
6	87.7

1.9 One pot "one step" vs one pot "two step"

Here we investigated whether it is advantageous to divide the reaction into two steps or whether the one-step method is more efficient. To do this, we ran the reaction for 1 hour at 25 Hz without adding hydrogen. The milling vessel was then gassed and milled for 2 hours at 35 Hz. The yields of this are shown in black for each step. The yield of the one-pot reaction are shown in green.

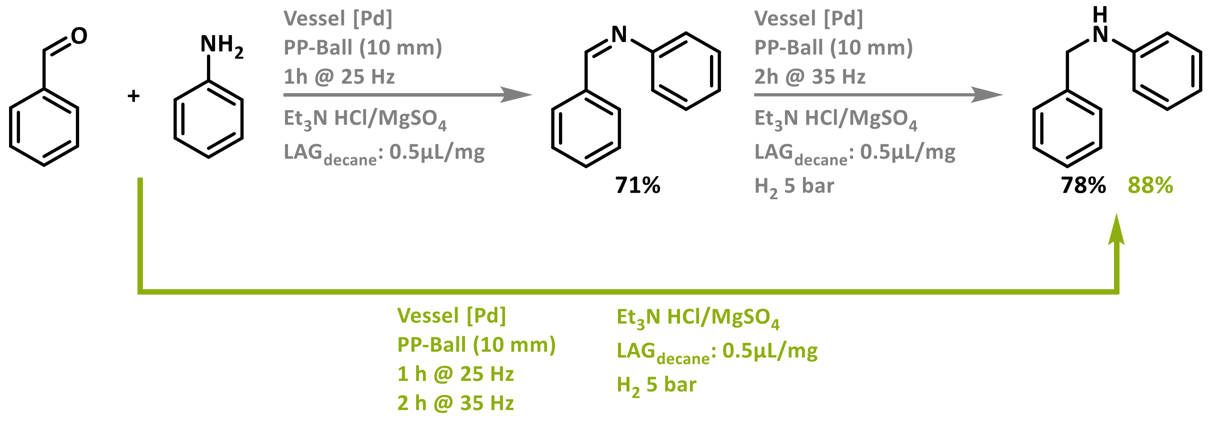

1.10 Coating calculations

Amount of Metal per L of electrolytes:

Electrolyte	Amount of metal per Liter / g
Copper	35
Nickel	30
Gold	5
Palladium	3

Needed amount of metal per coated ball:

Layer	Metal	Layerthickness / mm	Volume of metal / mm^3	Weight of metal per Layer / mg
1	Copper	0,00008	0,0754	0,675
2	Nickel	0,00012	0,1130	1,007
3	Gold	0,00006	0,0523	1,002
4	Palladium	0,00025	0,2357	2,833

If 1 L of each electrolyte is present, the limiting factor is the palladium electrolyte. Therefore, the number of balls that can be produced when 1 L of palladium electrolyte is present was calculated.

$$Number\ of\ Vessels = \frac{Amount\ of\ Pd\ per\ Liter}{Amount\ of\ needed\ Pd\ per\ Vessel} = \frac{3\ g}{0{,}002833\ g} = 1058{,}94$$

With 1 L of each electrolyte we are able to electroplate about 1050 vessels. This means about 1 mL electrolyte per liter “electrolyte” waste for each vessel, which is in total 4 mL (approx. 4 g).

Comparison to homogeneous system

A 1 mmol approach using 5 mol % of $Pd(PPh_3)_4$ as a standard catalyst means 57.8 mg need of catalyst. Since all reagents need to be solved we assume an amount of 5 mL solvent which means approx. 4-5 g waste.

Thus, in a single-use scenario, neither system can be considered sustainable.

However, our system has the advantage that catalyst separation and reuse are straightforward. In addition, working under ligand-free conditions avoids the need for air- and light-sensitive ligands, which often require the use of Schlenk techniques (where Argon is needed).

1.11 Screening of different milling parameters

The influence of various grinding parameters is examined below. The grinding conditions were kept constant and only the value shown was varied.

Bulk:

In order to investigate the influence of various bulk materials, different salts were used which do not participate in the reaction but can have a positive effect on it. Lithium salts were deliberately avoided, as previous work has shown that this leads to the preferential formation of alcohol.

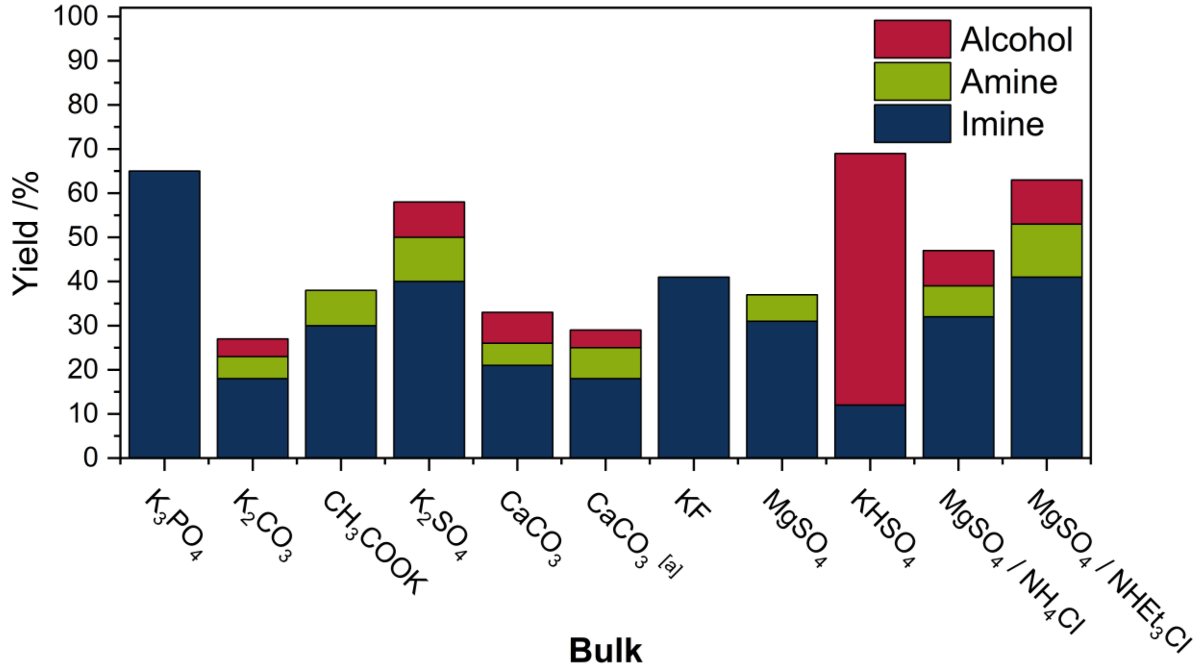

Figure S2: Screening of different Bulk materials. Reaction conditions: 1 mmol benzaldehyde, 2 mmol aniline, 1 g of bulk material were milled with one 10 mm polypropylene ball in a 19 mL palladium-coated steel gas vessel for 2 h at 30 Hz in a MM500 at 5 bar H_2, LAG: Decane with η=0.5 µL/mg.[a] $CaCO_3$ obtained from cleaned and ground eggshell waste.

Frequency:

Here, the grinding frequencies at which by-product formation is lowest and the yield of the imine intermediate is highest were investigated.

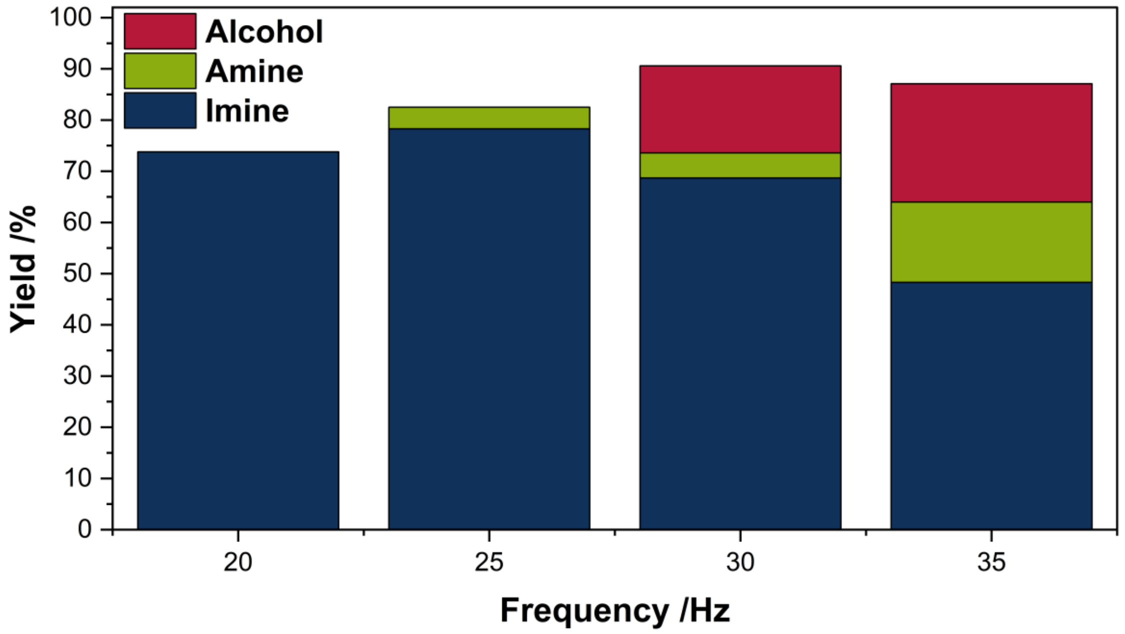

Figure S3: Influence of different milling frequencies. 1 mmol benzaldehyde, 2 mmol aniline, 1 g of bulk material (50:50 magnesium sulfate:Triethylamine hydrochloride) were milled with one 10 mm polypropylene ball in a 19 mL palladium-coated steel gas vessel for 2 h at 30 Hz in a MM500 at 5 bar H_2, LAG: Decane with η=0.5 µL/mg.

In the following figure, the frequency has been optimized for the wide step. Previously, the reaction ran for 1 hour at 25 Hz to optimize hydrogenation.

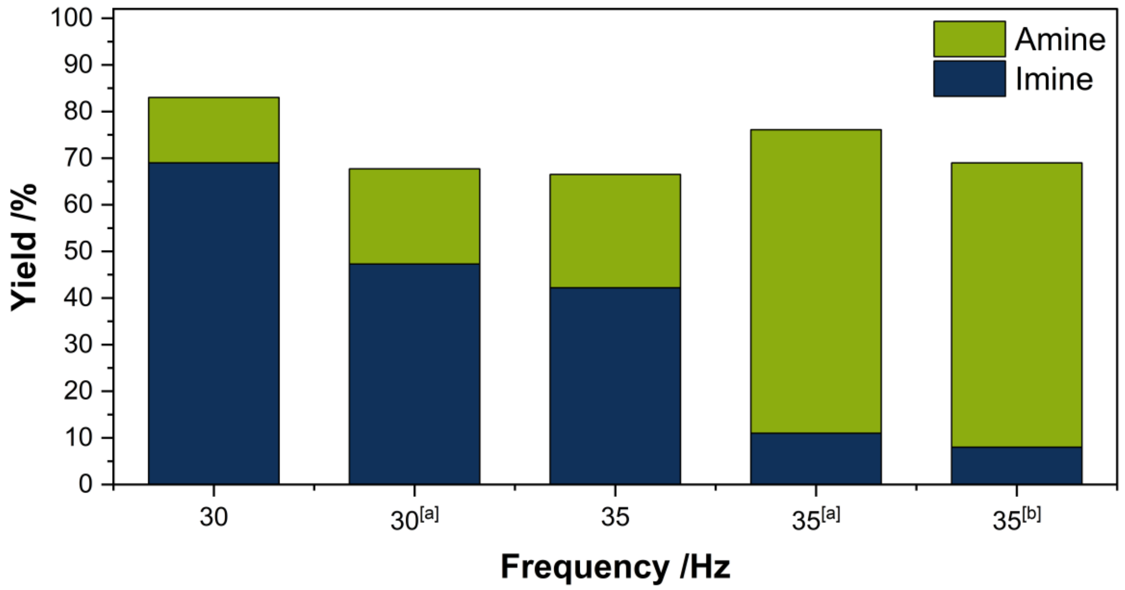

Figure S4: Screening of the milling frequency for the second step after 2h at 25 Hz. 1 mmol benzaldehyde, 2 mmol aniline, 1 g of bulk material (0.5 g magnesium sulfate and 0.5 g triethylamine hydrochloride) were milled with one 10 mm polypropylene ball in a 19 mL palladium-coated steel gas vessel for 2 h at 25 Hz and 1 h at different frequencies for the second step in a MM500 at 5 bar H_2, LAG: Decane with η=0.5 µL/mg. [a] 2 h, [b] 3 h

LAG:

In this section, the influence of various liquid additives was investigated. Solvents with different polarities and densities were examined, which cannot participate directly in the reaction. Furthermore, the amount of solvent was optimized.

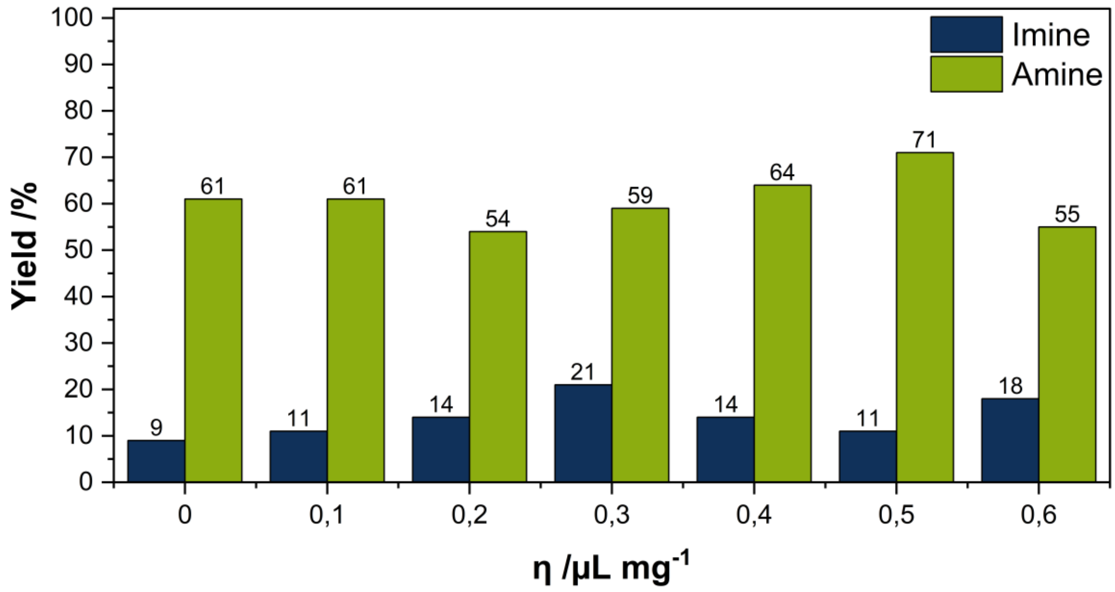

Figure S5: influence of the LAG amount. 1 mmol benzaldehyde, 2 mmol aniline, 1 g of bulk material (0.5 g magnesium sulfate and 0.5 g triethylamine hydrochloride) were milled with one 10 mm polypropylene ball in a 19 mL palladium-coated steel gas vessel for 1 h at 25 Hz and 2 h at 35 Hz in a MM500 at 5 bar H_2, LAG: Decane

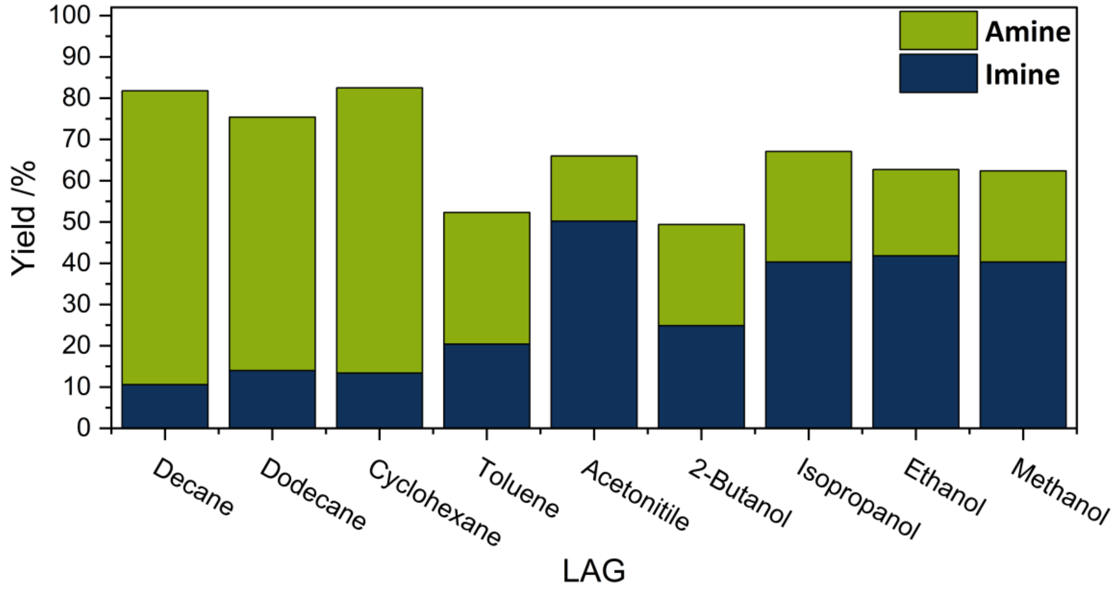

Figure S6: influence of different LAG agents. 1 mmol benzaldehyde, 2 mmol aniline, 1 g of bulk material (0.5 g magnesium sulfate and 0.5 g triethylamine hydrochloride) were milled with one 10 mm polypropylene ball in a 19 mL palladium-coated steel gas vessel for 1 h at 25 Hz and 2 h at 35 Hz in a MM500 at 5 bar H_2, with η=0.5 µL/mg

2. Characterization

2.1. Products

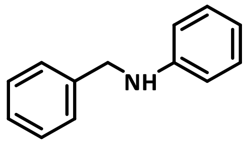

Benzylanilin

^{1}H NMR (400 MHz, $CDCl_3$) δ 7.41 – 7.33 (m, 4H), 7.33 – 7.22 (m, 2H), 7.22 – 7.12 (m, 2H), 6.71 (tt, *J* = 7.3, 1.1 Hz, 1H), 6.67 – 6.59 (m, 2H), 4.32 (s, 2H).

The NMR data agree with the values reported in the literature (cf. Ref. S1).

EI-MS (*m/z*): [M+] calc. 183.1 found: 183

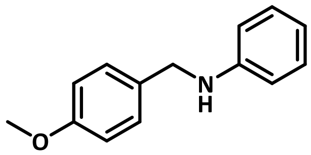

N-(4-Methoxybenzyl)aniline

^{1}H NMR (400 MHz, $CDCl_3$) δ 7.35 – 7.27 (m, 2H), 7.20 – 7.13 (m, 2H), 6.92 – 6.84 (m, 2H), 6.71 (m, 1H), 6.67 – 6.60 (m, 2H), 4.25 (s, 2H), 3.80 (s, 3H).

The NMR data agree with the values reported in the literature (cf. Ref. S2).

EI-MS (*m/z*): [M+] calc. 213.5 found:213

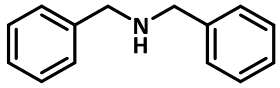

Dibenzylamin

^{1}H NMR (400 MHz, $CDCl_3$) δ 7.51 – 7.17 (m, 10H), 3.92 – 3.80 (m, 4H).

The NMR data agree with the values reported in the literature (cf. Ref. S1).

EI-MS (*m/z*): [M+] calc. 197.3 found: 196

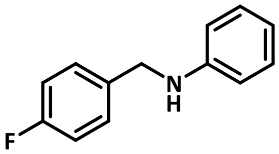

N-(4-Fluorobenzyl)aniline

^{1}H NMR (400 MHz, $CDCl_3$) δ 7.38 – 7.29 (m, 2H), 7.22 – 7.14 (m, 2H), 7.09 – 6.99 (m, 2H), 6.78 – 6.69 (m, 1H), 6.68 – 6.60 (m, 2H), 4.31 (s, 2H).

The NMR data agree with the values reported in the literature (cf. Ref. S2).

EI-MS (*m/z*): [M+] calc. 201.2 found: 201

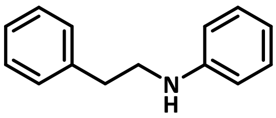

N-Phenethylaniline

^{1}H NMR (400 MHz, $CDCl_3$) δ 7.32 – 7.14 (m, 7H), 6.81 (m, 1H), 6.62 – 6.56 (m, 2H), 3.45 (m, 2H), 2.90 (t, 2H).

The NMR data agree with the values reported in the literature (cf. Ref. S1).

EI-MS (*m/z*): [M+] calc. 197.3 found: 197

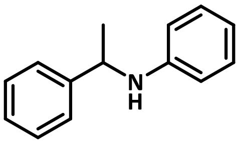

N-(1-Phenylethyl)aniline

^{1}H NMR (400 MHz, Chloroform-*d*) δ 7.33 – 7.24 (m, 5H), 7.10 – 7.03 (m, 2H), 6.81 (m, 1H), 6.65 – 6.59 (m, 2H), 4.56 (m, 1H), 1.51 (s, 3H).

The NMR data agree with the values reported in the literature (cf. Ref. S3).

EI-MS (*m/z*): [M+] calc. 197.3 found: 197

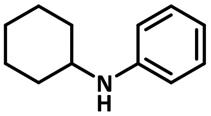

N-cyclohexylaniline

^{1}H NMR (400 MHz, $CDCl_3$) δ 7.19 – 7.10 (m, 2H), 6.69 – 6.63 (m, 1H), 6.63 – 6.53 (m, 2H), 3.24 (tt, *J* = 10.2, 3.8 Hz, 1H), 2.04 (dt, *J* = 12.3, 3.8 Hz, 2H), 1.81 – 1.69 (m, 2H), 1.69 – 1.58 (m, 1H), 1.43 – 1.06 (m, 5H).

The NMR data agree with the values reported in the literature (cf. Ref. S4).

EI-MS (*m/z*): [M+] calc. 175.1 found: 175

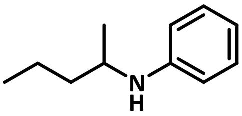

N-(Pentan-2-yl)aniline

^{1}H NMR (400 MHz, Chloroform-*d*) δ 7.08 – 7.01 (m, 2H), 6.84 – 6.77 (m, 1H), 6.69 – 6.63 (m, 2H), 3.53 – 3.51 (m, 1H), 1.69 – 1.59 (m, 1H), 1.47 – 1.33 (m, 2H), 1.33 – 1.20 (m, 1H), 1.19 – 1.11 (m, 3H), 0.96 – 0.87 (m, 3H).

The NMR data agree with the values reported in the literature (cf. Ref S5).

EI-MS (m/z): [M+] calc. 163.3 found: 163

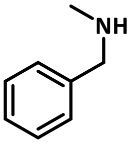

N-Methylbenzylamine

^{1}H NMR (400 MHz, $CDCl_3$) δ 7.41 – 7.19 (m, 5H), 3.73 (s, 2H), 2.44 (s, 3H).

The NMR data agree with the values reported in the literature (cf. Ref. S6).

EI-MS (m/z): [M+] calc. 121.1 found: 121

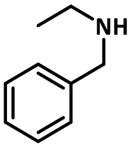

N-Ethylbenzylamine

^{1}H NMR (400 MHz, $CDCl_3$) δ 7.36 – 7.18 (m, 5H), 3.78 (s, 2H), 2.72 – 2.62 (m, 2H), 1.12 (t, *J* = 7.1 Hz, 3H).

The NMR data agree with the values reported in the literature (cf. Ref S6).

EI-MS (m/z): [M+] calc. 135.1 found: 135

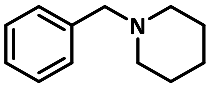

Benzylpiperidine

^{1}H NMR (400 MHz, $CDCl_3$) δ 7.35 – 7.19 (m, 5H), 3.47 (s, 2H), 2.37 (d, *J* = 10.3 Hz, 4H), 1.57 (p, *J* = 5.6 Hz, 4H), 1.43 (h, *J* = 4.8 Hz, 2H).

The NMR data agree with the values reported in the literature (cf. Ref. S7).

EI-MS (m/z): [M+] calc. 175.1 found: 175

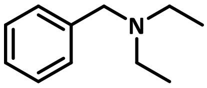

Benzyldiethylamine

^{1}H NMR (400 MHz, $CDCl_3$) δ 7.37 – 7.18 (m, 5H), 3.56 (s, 2H), 2.52 (q, *J* = 7.1 Hz, 4H), 1.04 (t, *J* = 7.1 Hz, 6H).

The NMR data agree with the values reported in the literature (cf. Ref. S8).

EI-MS (m/z): [M+] calc. 163.2 found: 163

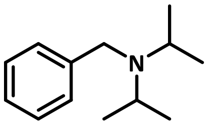

Benzyldiisopropylamine

^{1}H NMR (400 MHz, $CDCl_3$) δ 7.43 – 7.35 (m, 2H), 7.34 – 7.15 (m, 3H), 3.68 – 3.62 (m, 2H), 3.09 – 2.94 (m, 2H), 1.03 (ddd, *J* = 6.6, 5.0, 2.9 Hz, 12H).

The NMR data agree with the values reported in the literature (cf. Ref S7).

EI-MS (m/z): [M+] calc. 191.3 found: 192

2.2 Mass Spectra

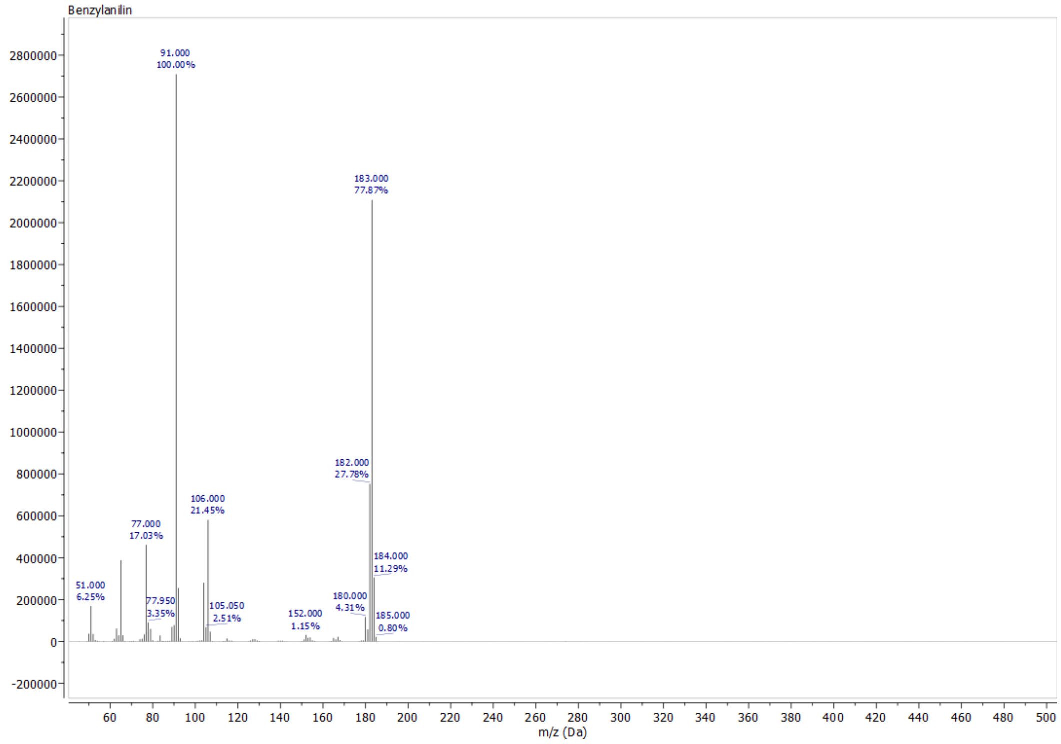

Figure S7: EI-MS of Benzylamine.

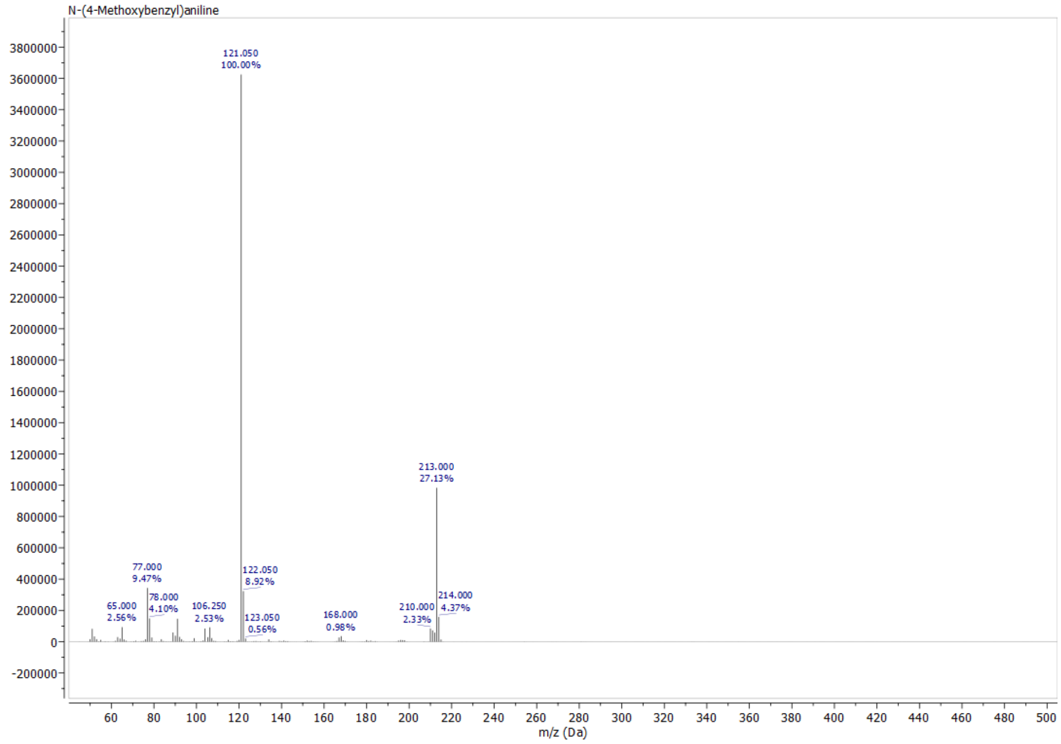

Figure S8: EI-MS of N-(4-Methoxybenzyl)aniline.

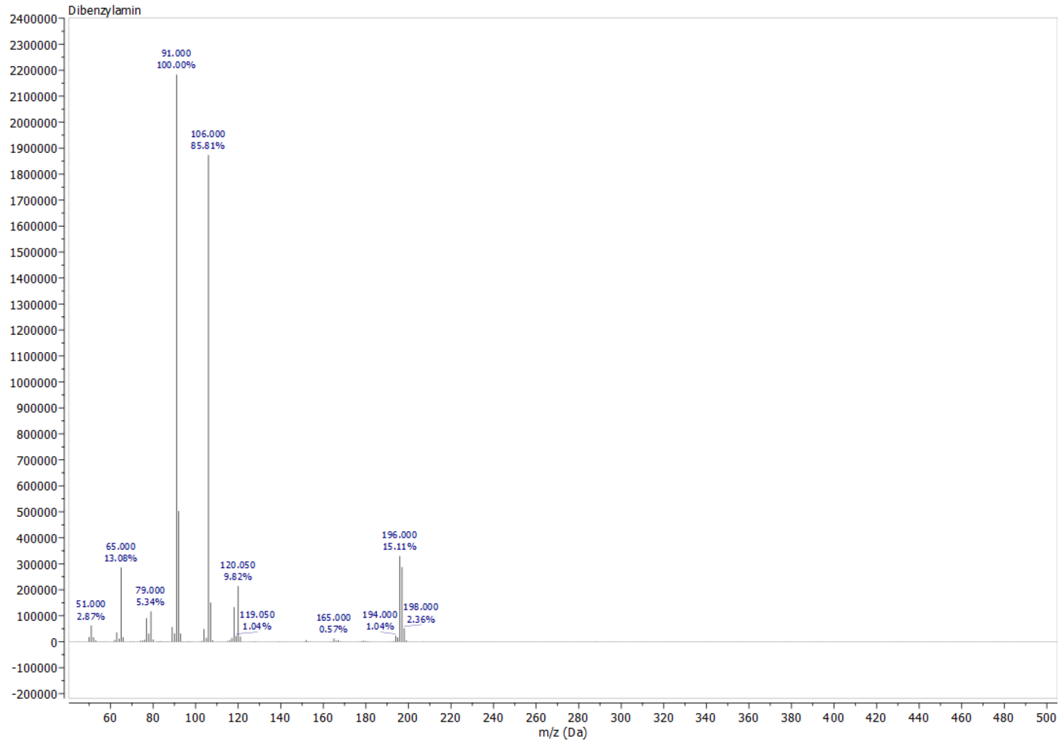

Figure S9: EI-MS of Dibenzylamine.

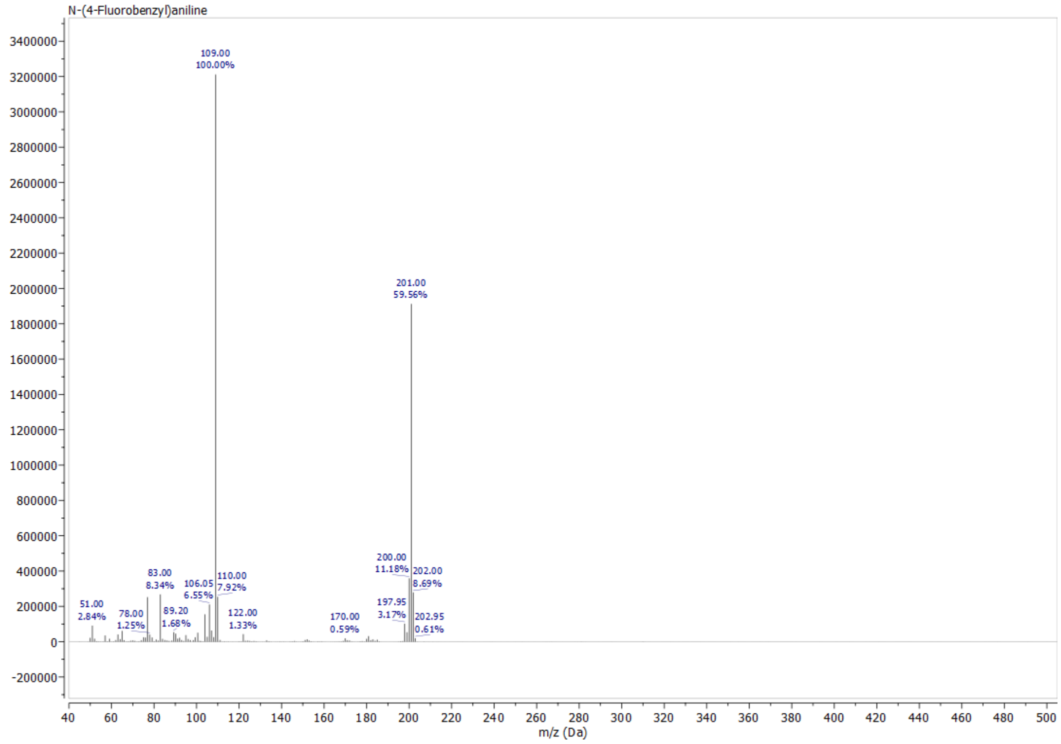

Figure S10S: EI-MS of N-(4-Fluorobenzyl)aniline.

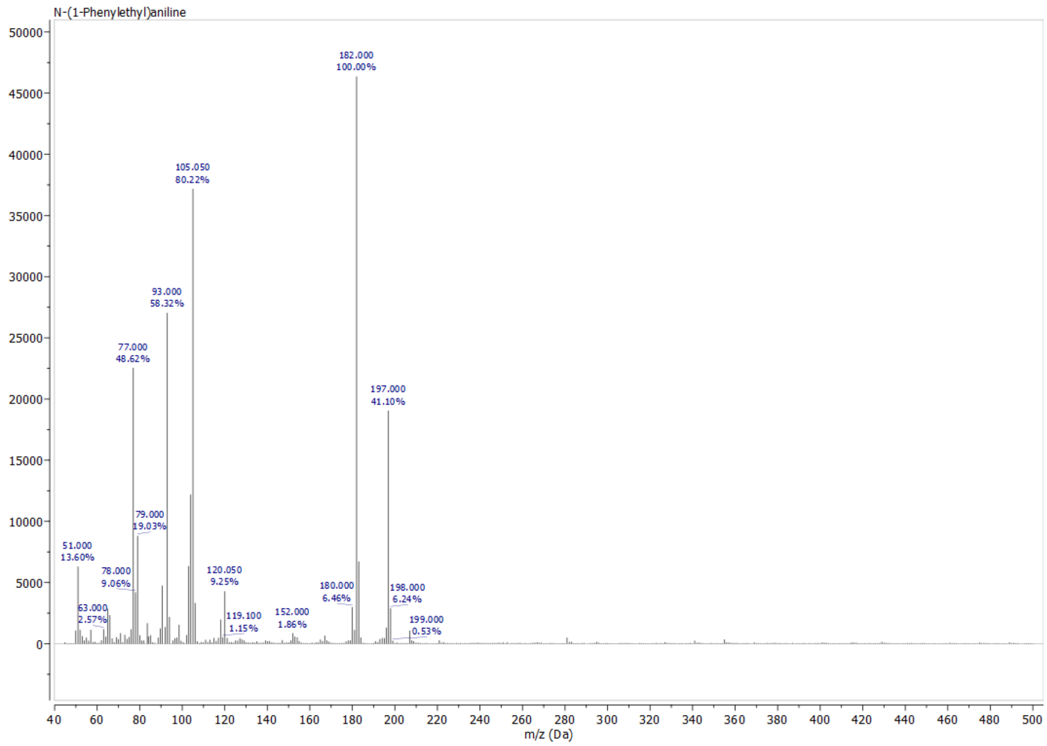

Figure 11:EI-MS of N-Phenethylaniline.

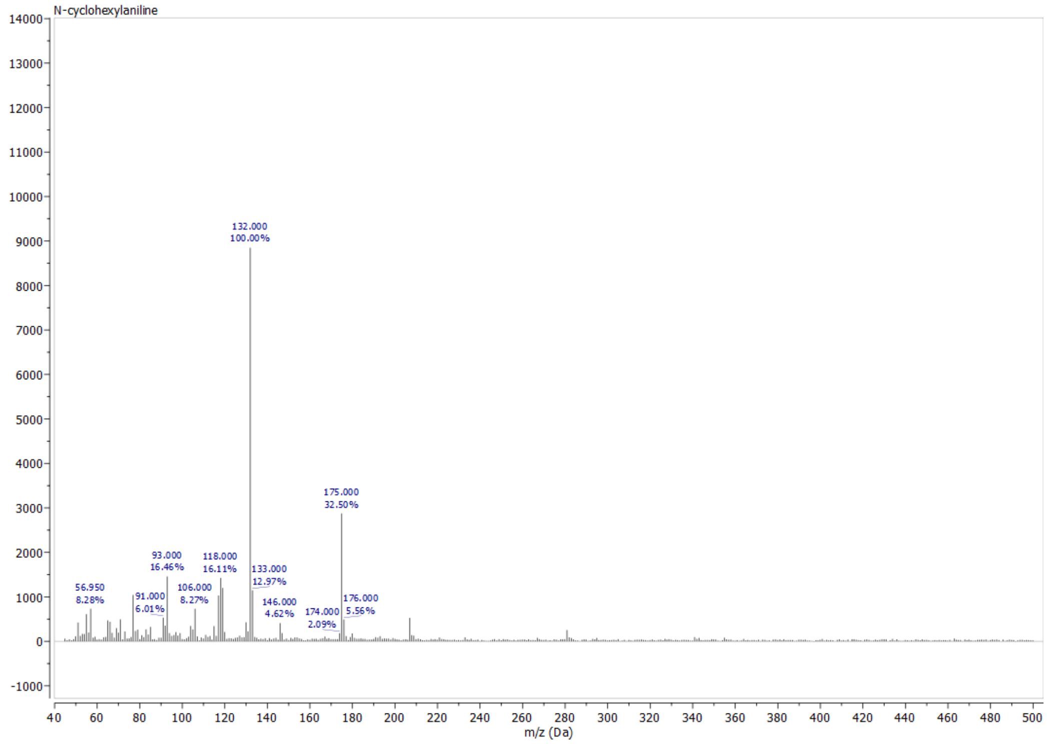

Figure S12: EI-MS of N-Cyclohexylanilin.

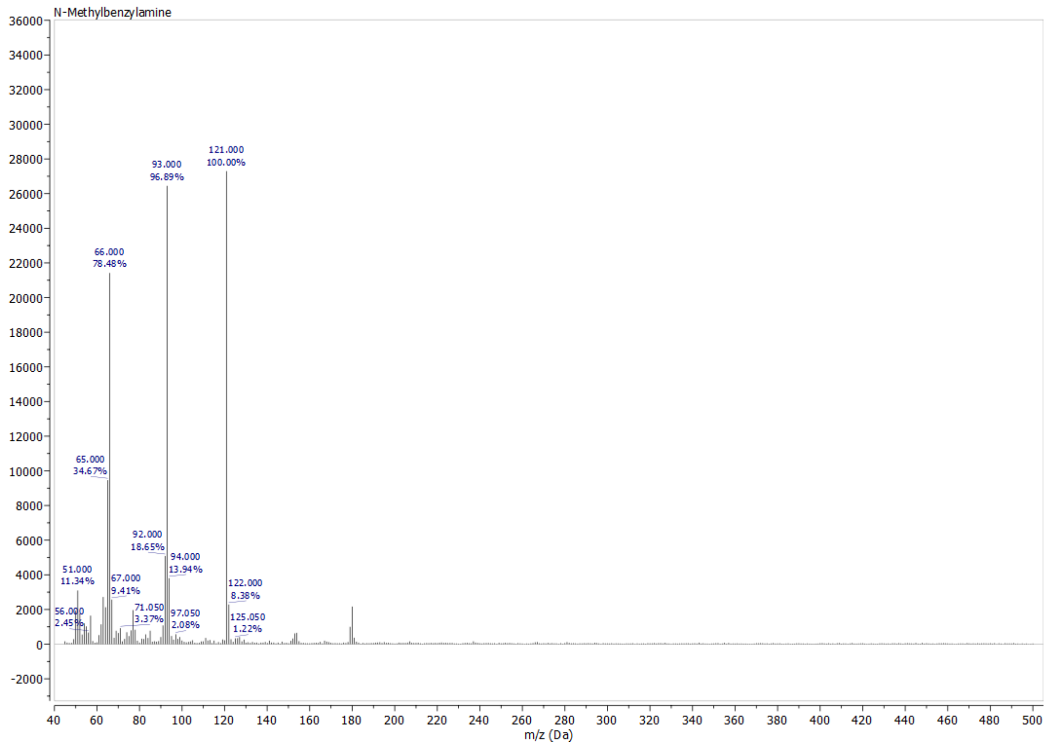

Figure S13: EI-MS of N-Methylbenzylamine.

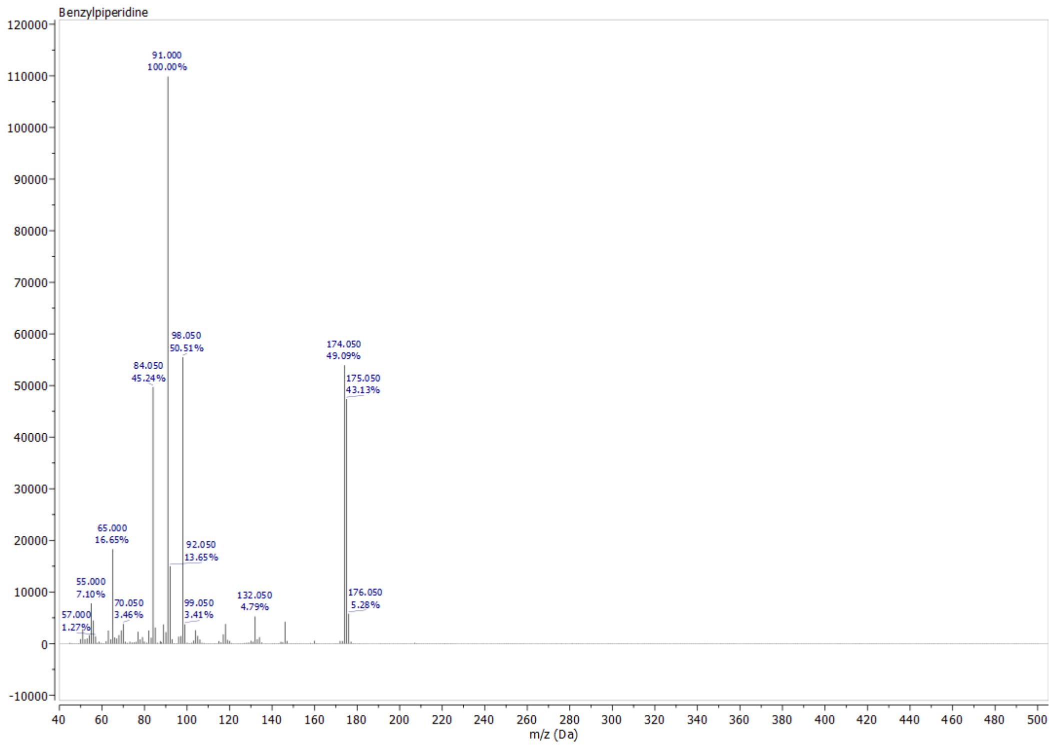

Figure S14: EI-MS of Benzylpiperidine.

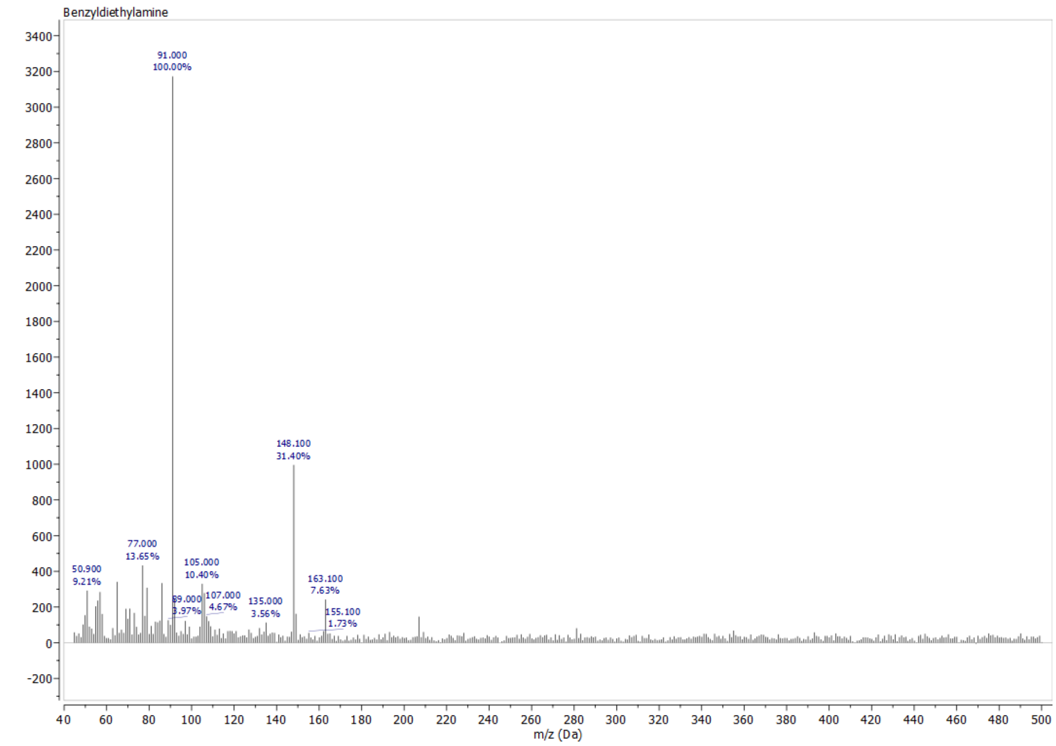

Figure S15: EI-MS of Benzyldiethylamine.

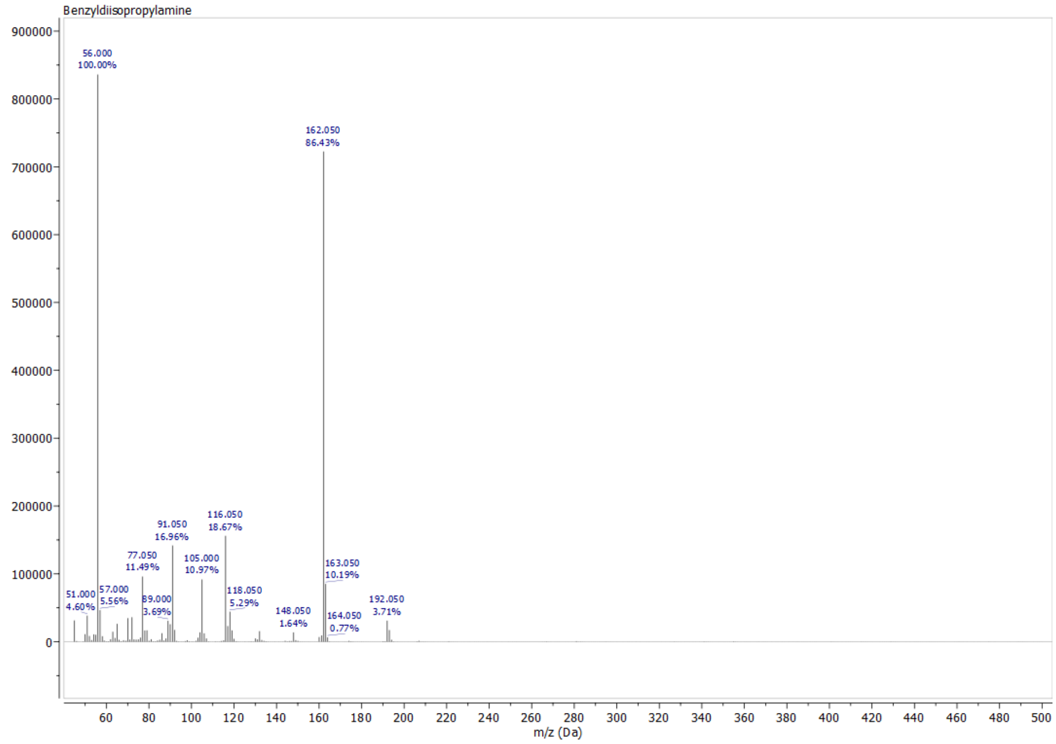

Figure S16: EI-MS of Benzyldiisopropylamine.

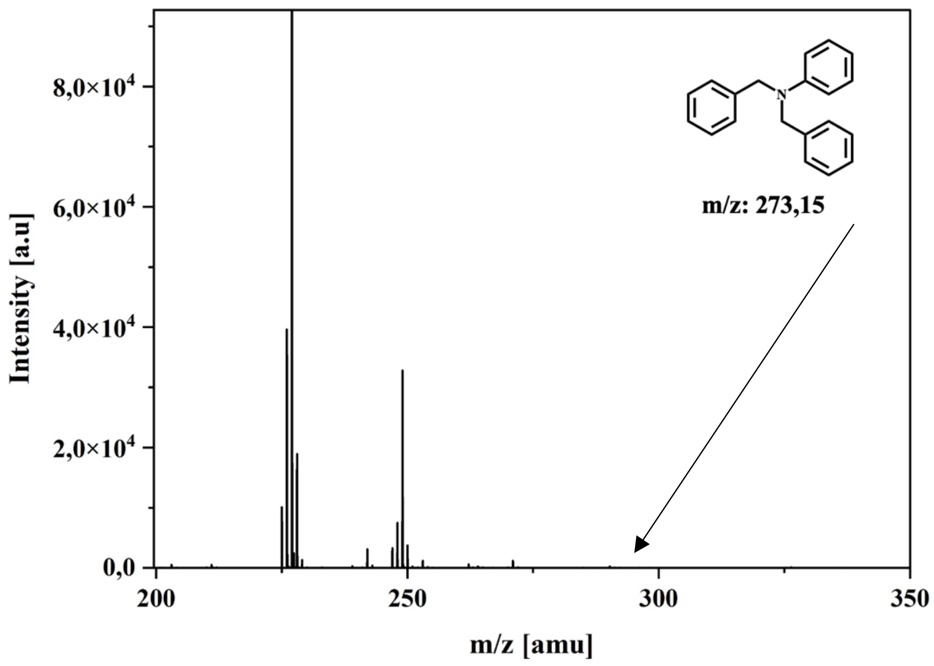

Figure S17: Maldi-TOF of the tertiary amine product generated by overalkylation.

2.3 NMR

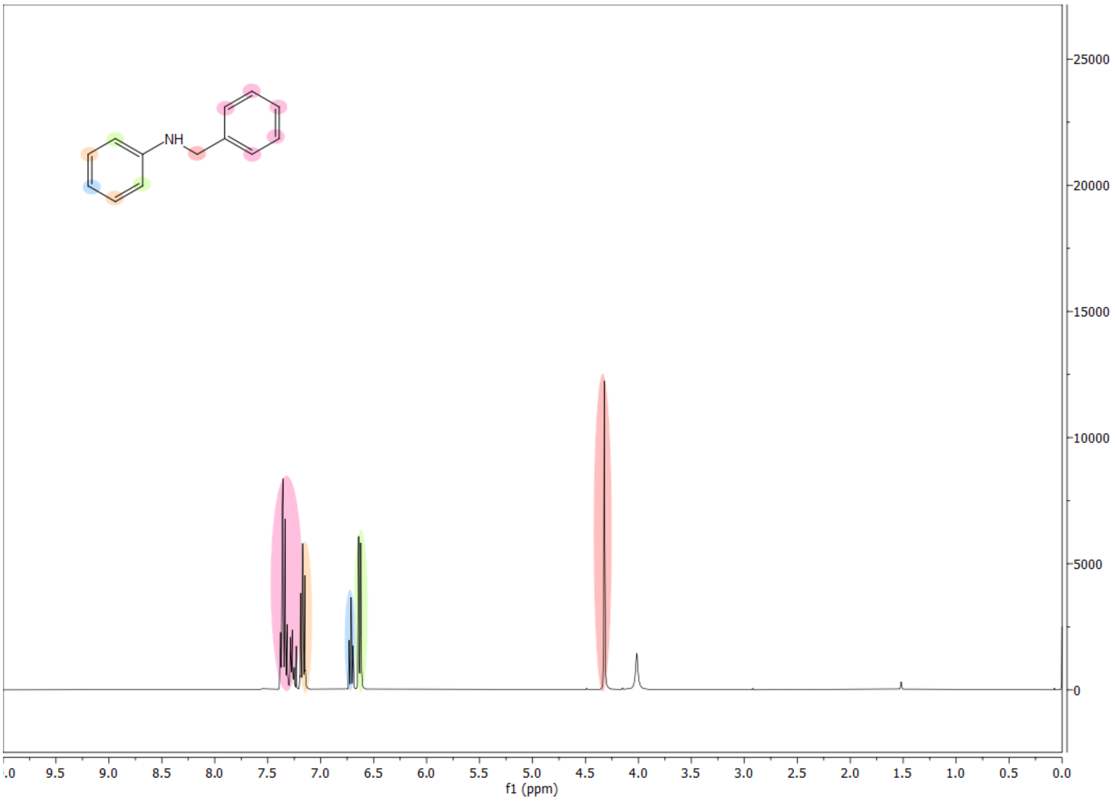

Figure S18: ^{1}H-NMR of Benzylanilin

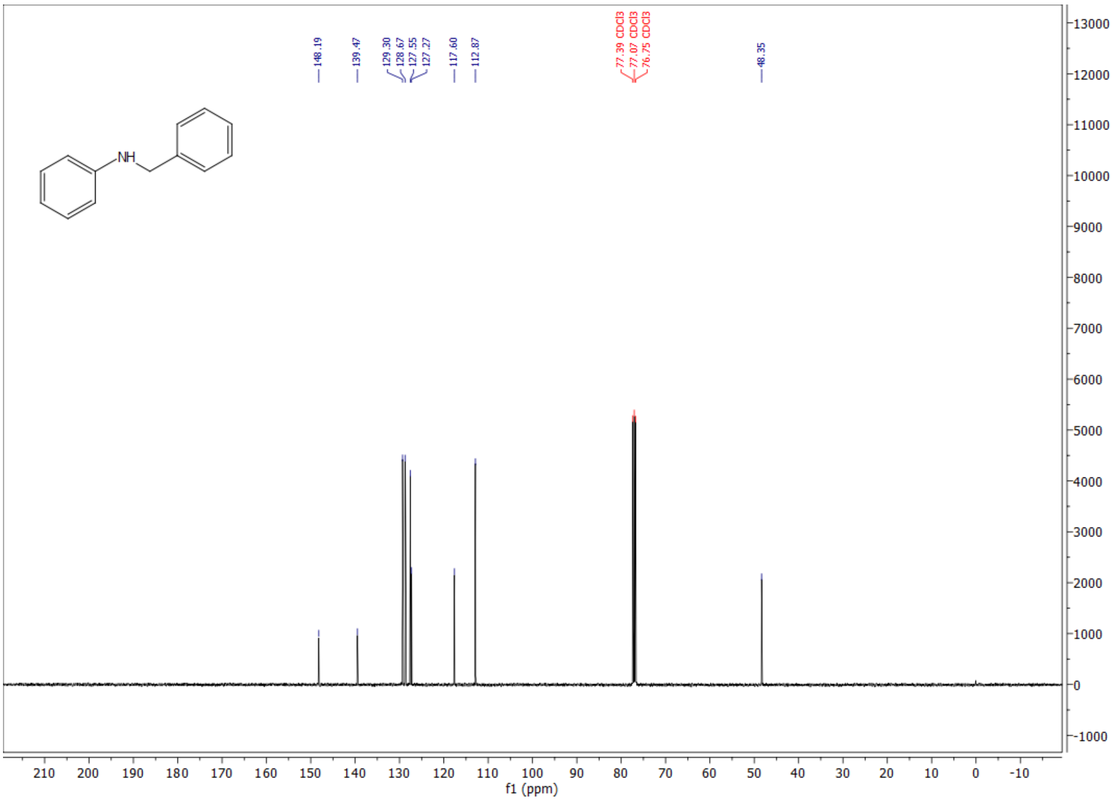

Figure S19: ^{13}C-NMR of Benzylaniline.

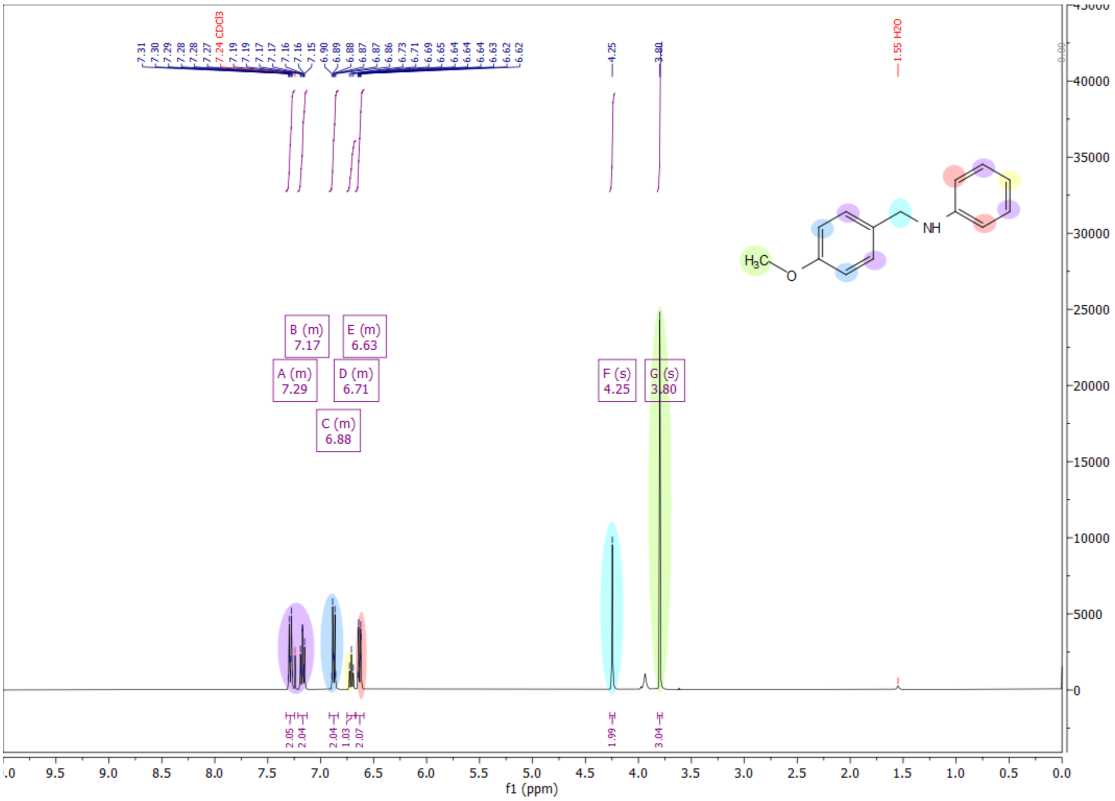

Figure S20: ^{1}H-NMR of N-(4-Methoxybenzyl)aniline

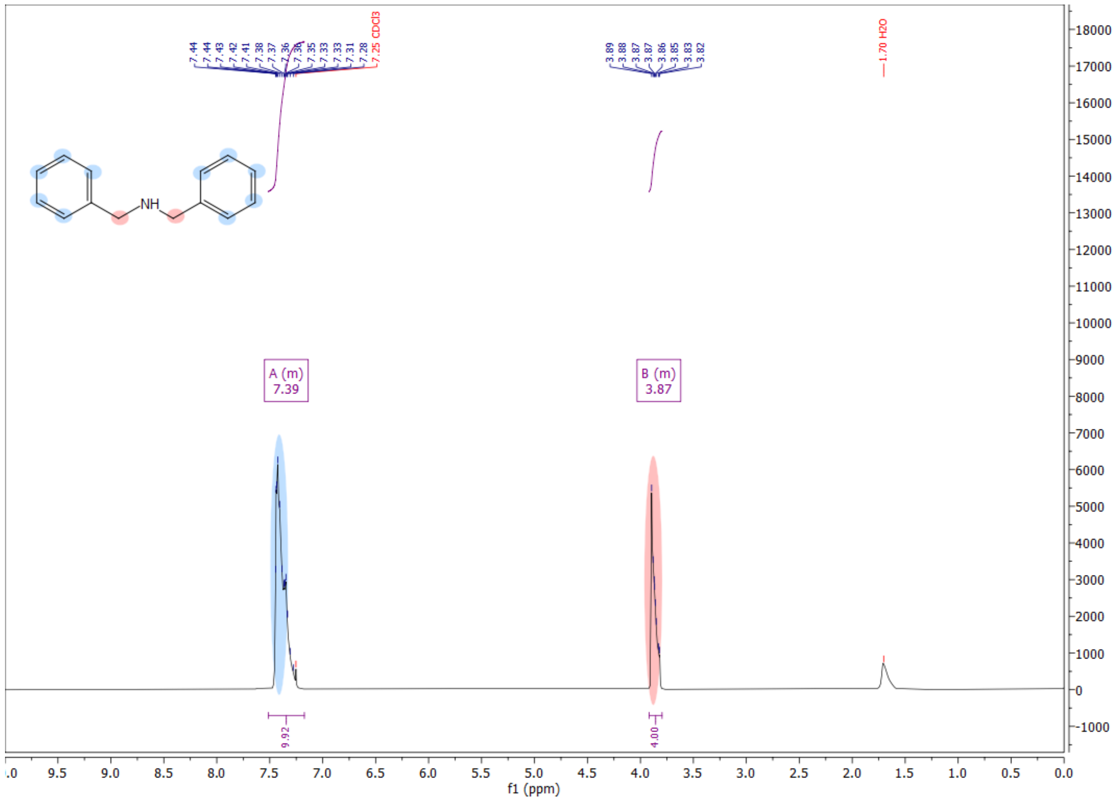

Figure S21: ^{1}H-NMR of Dibenzylamin

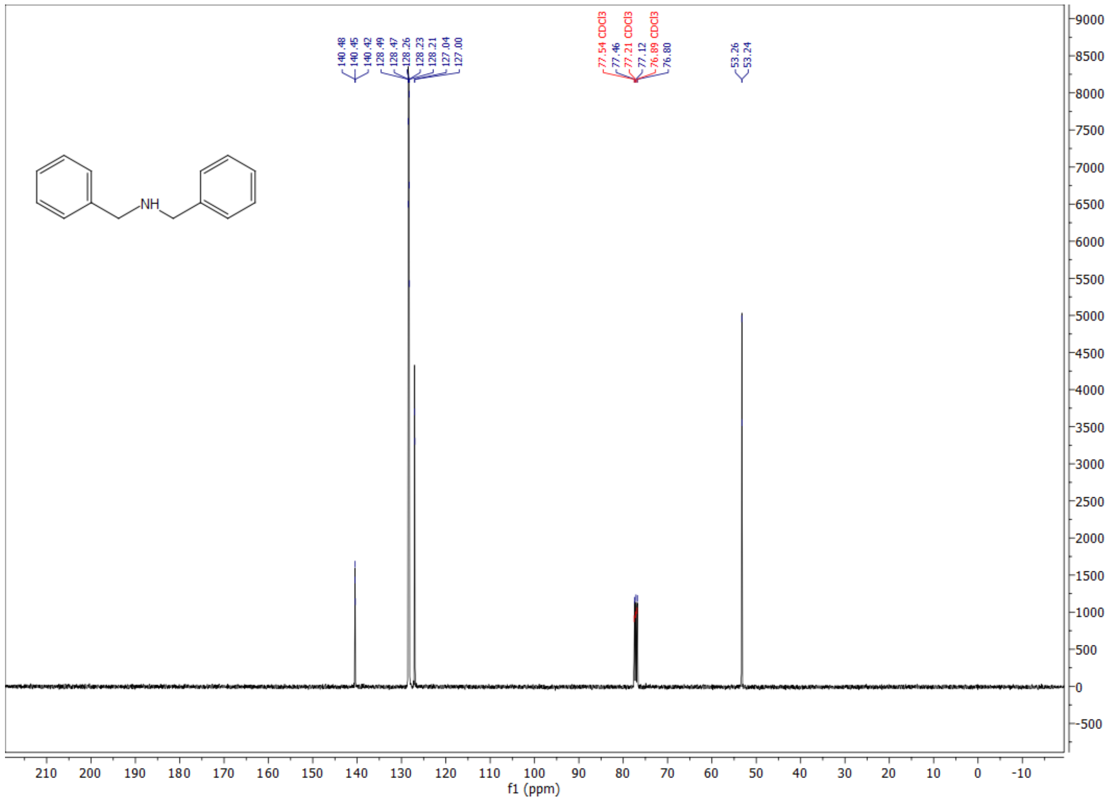

Figure S22: ^{13}C-NMR of Dibenzylamin.

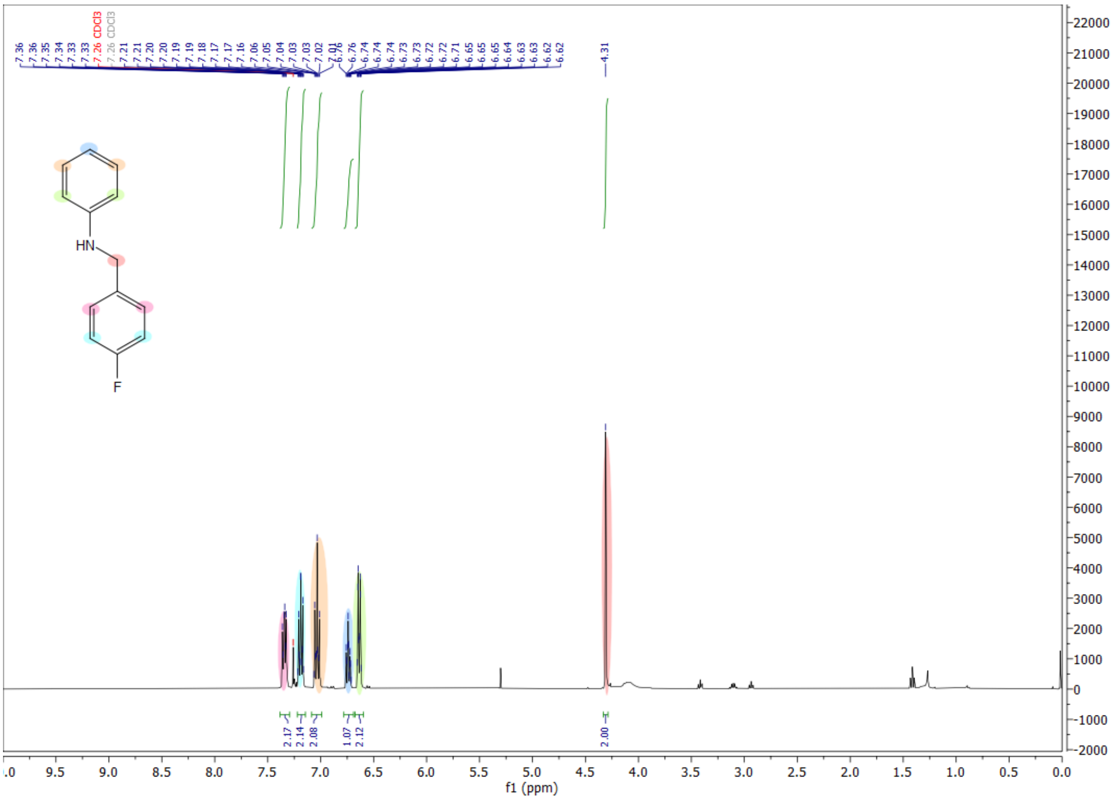

Figure S23: ^{1}H-NMR of N-(4-Fluorobenzyl)aniline.

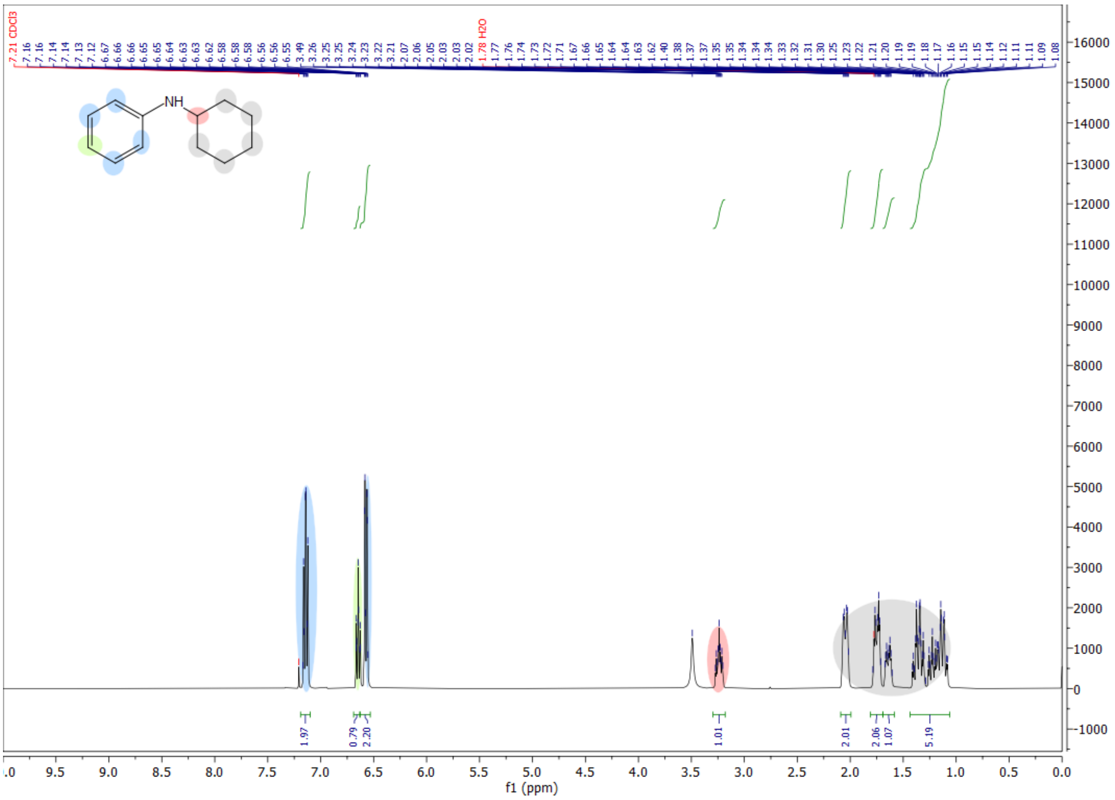

Figure S24: ^{1}H-NMR of Cyclohexylanilin.

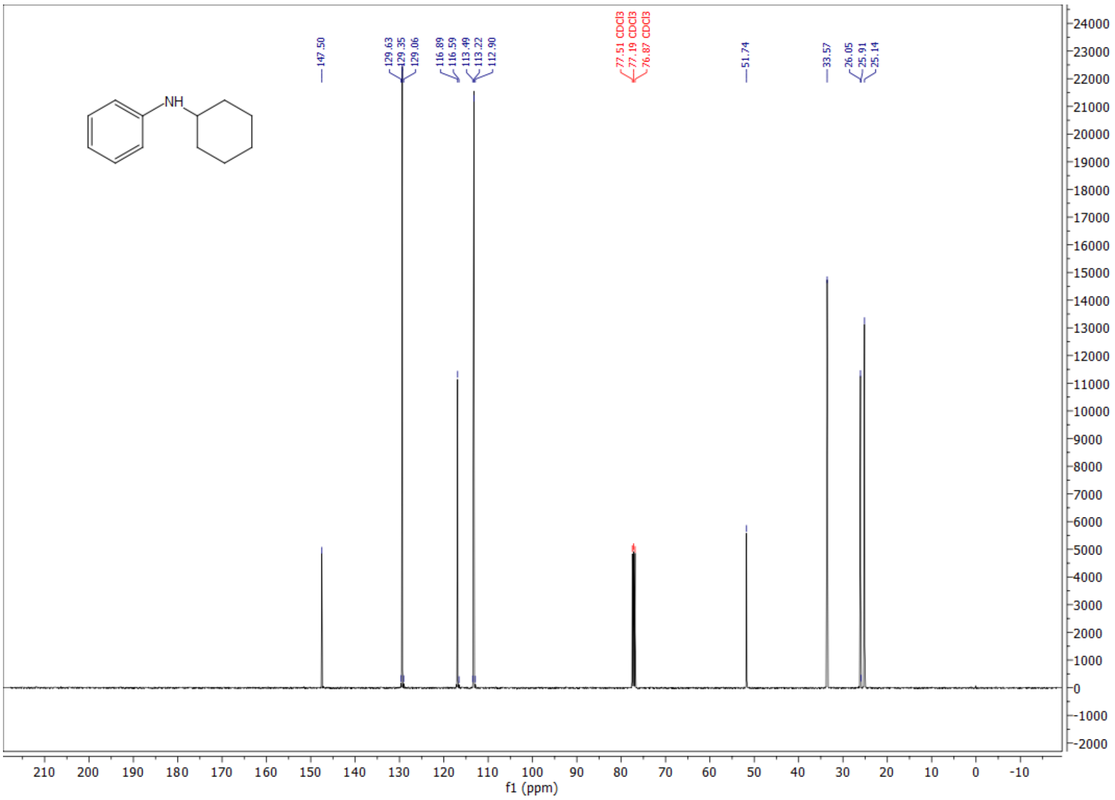

Figure S25: ^{13}C-NMR of N-Cyclohexylanilin.

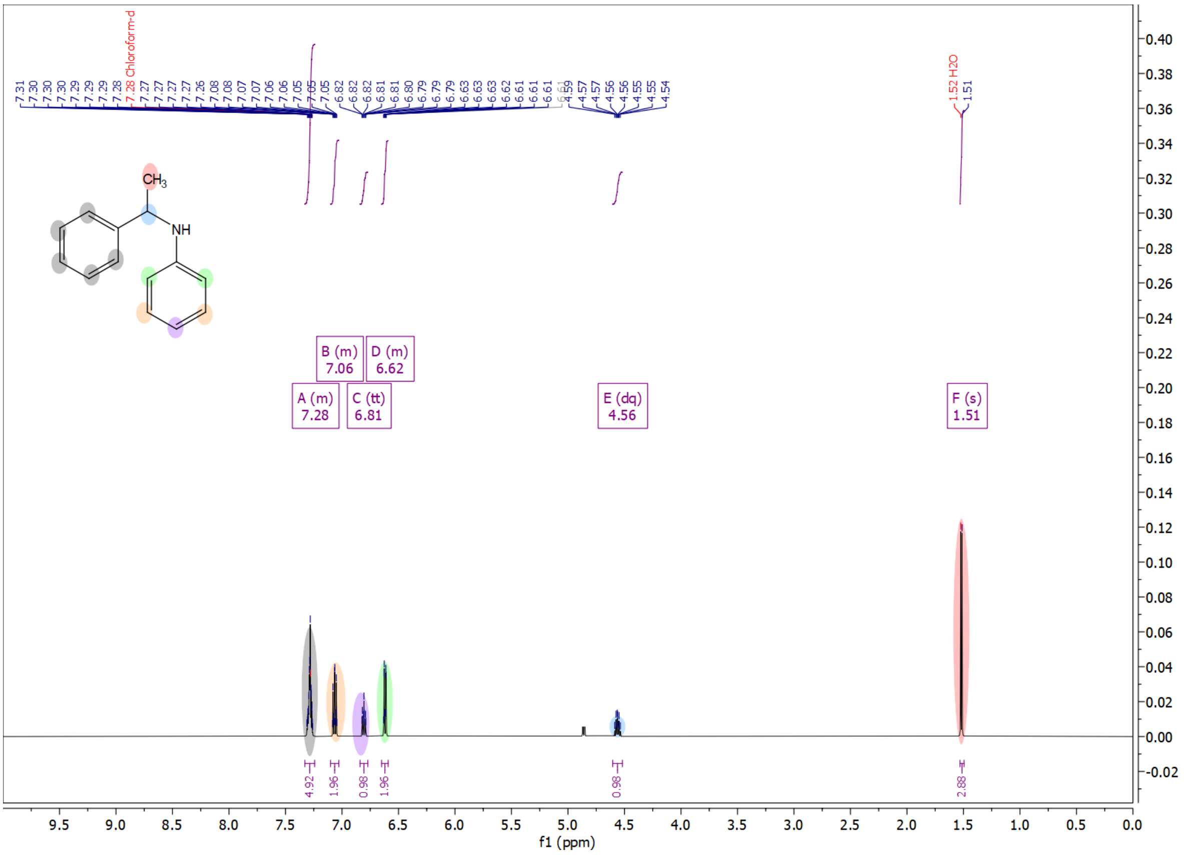

Figure 26: ^{1}H-NMR of N-(1-Phenylethyl)aniline

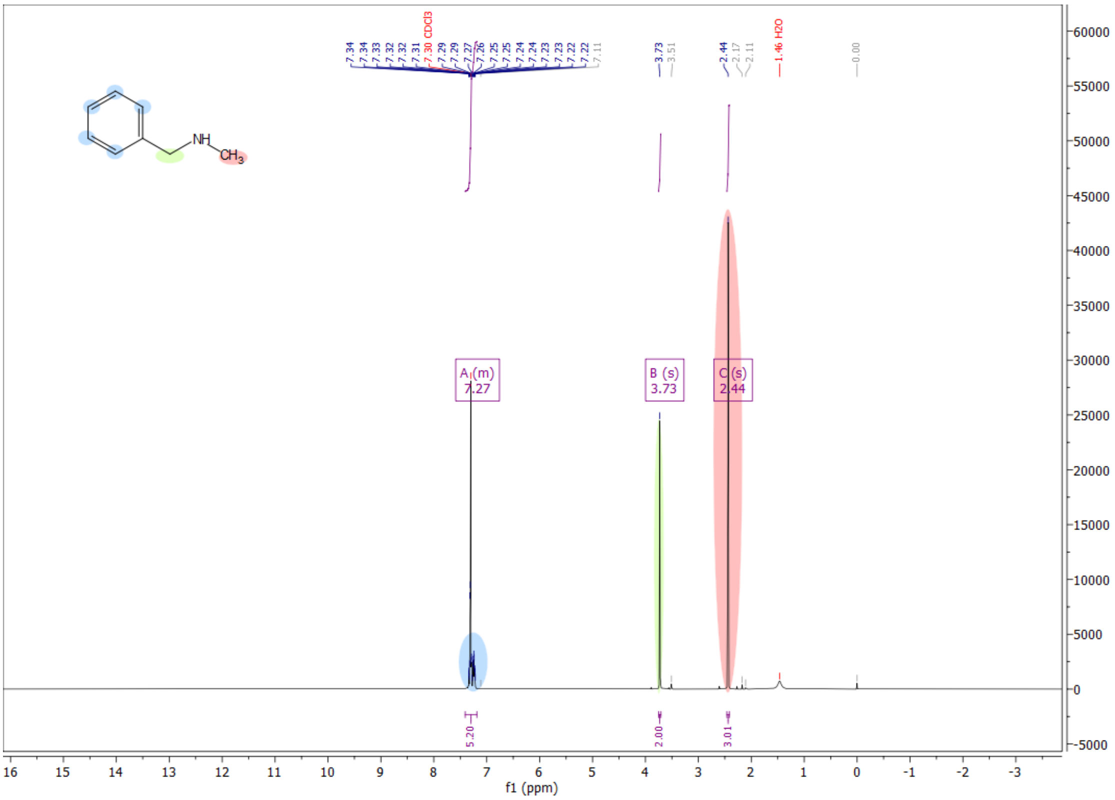

Figure S27: ^{1}H-NMR of N-Methylbenzylamine

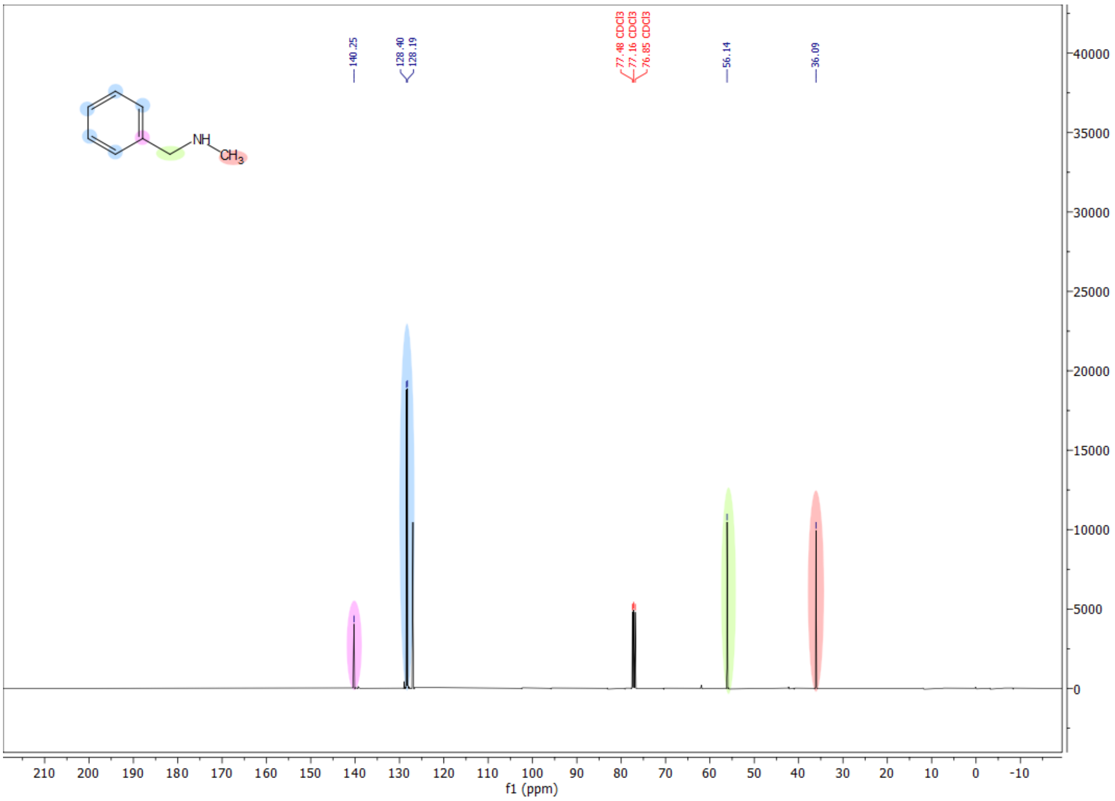

Figure S28: ^{13}C-NMR of N-Methylbenzylamine.

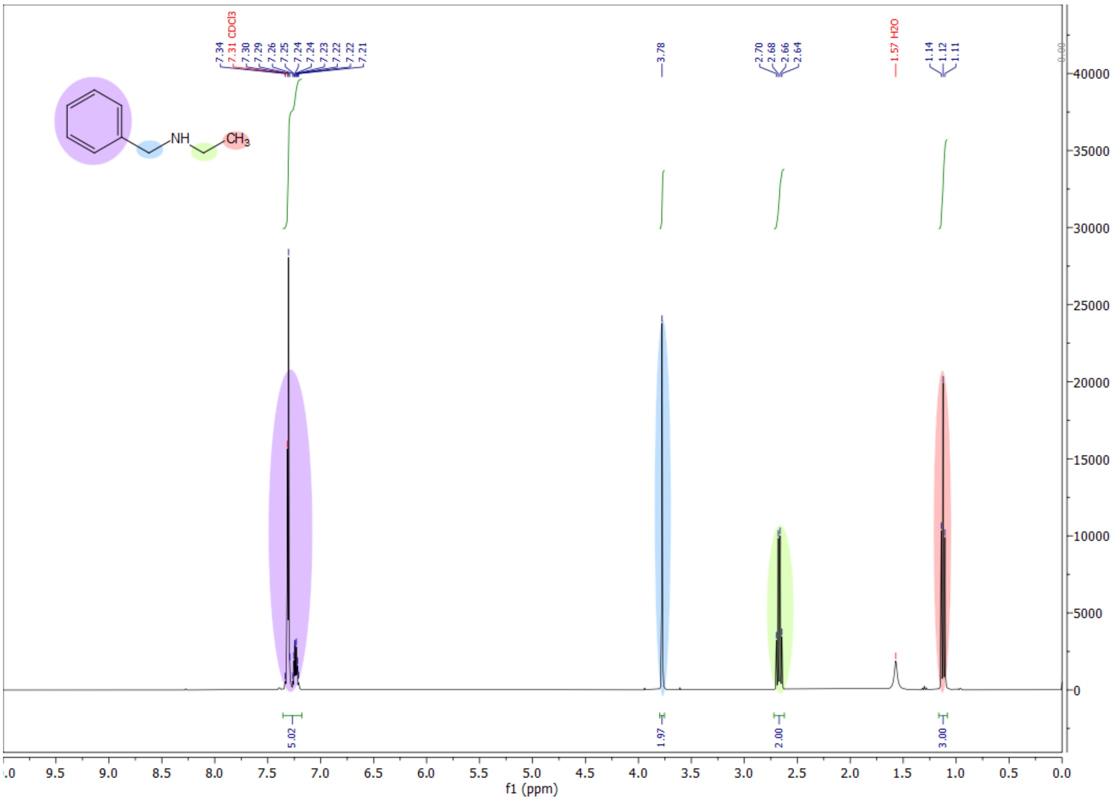

Figure S29: ^{1}H-NMR of N-Ethylbenzylamine

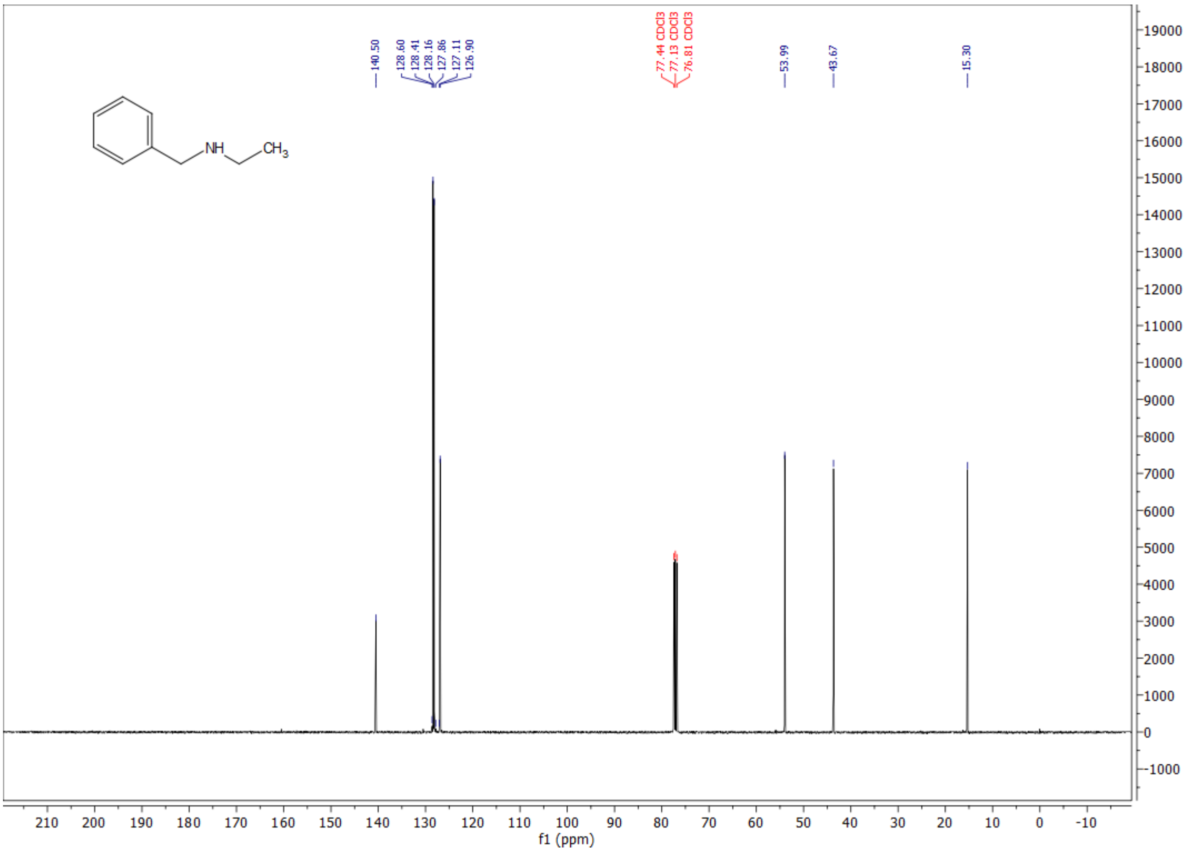

Figure S30: ^{13}C-NMR of N-Ethylbenzylamine

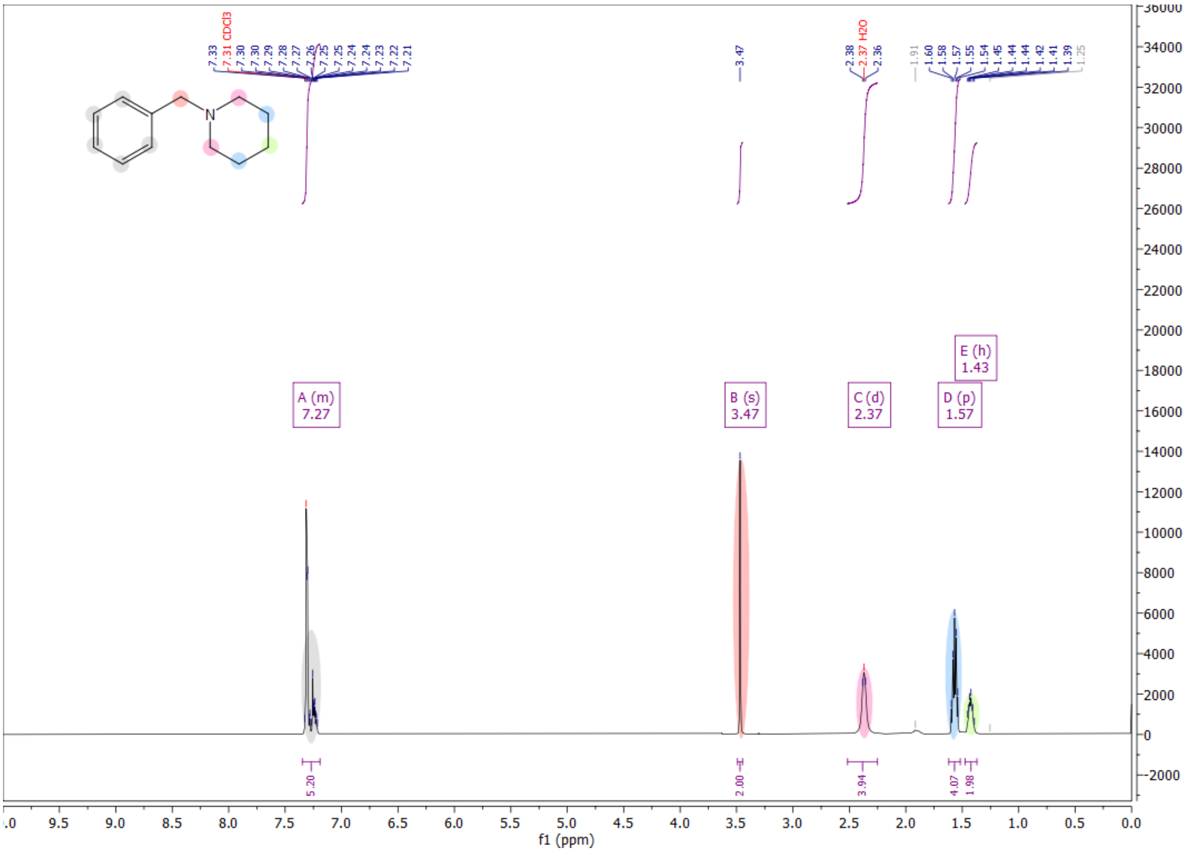

Figure S31: ^{1}H-NMR of Benzylpiperidine.

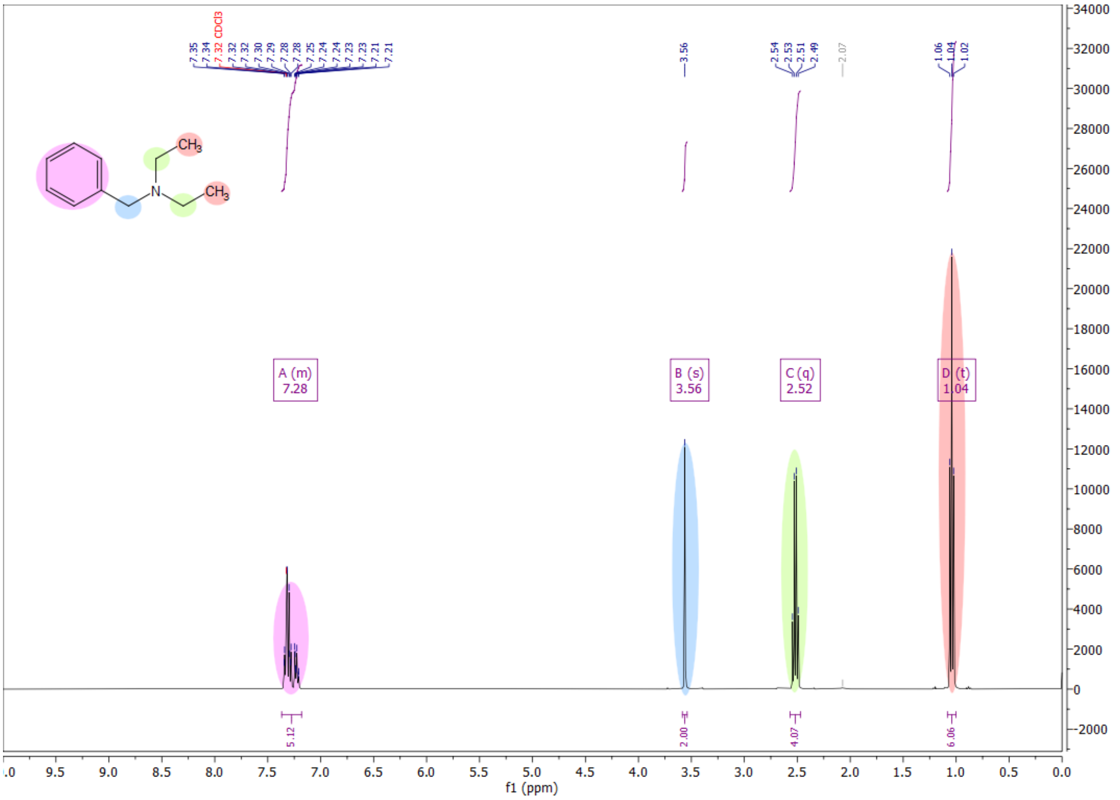

Figure S32: ^{1}H-NMR of N-Benzyl-N-Ethylethanamin

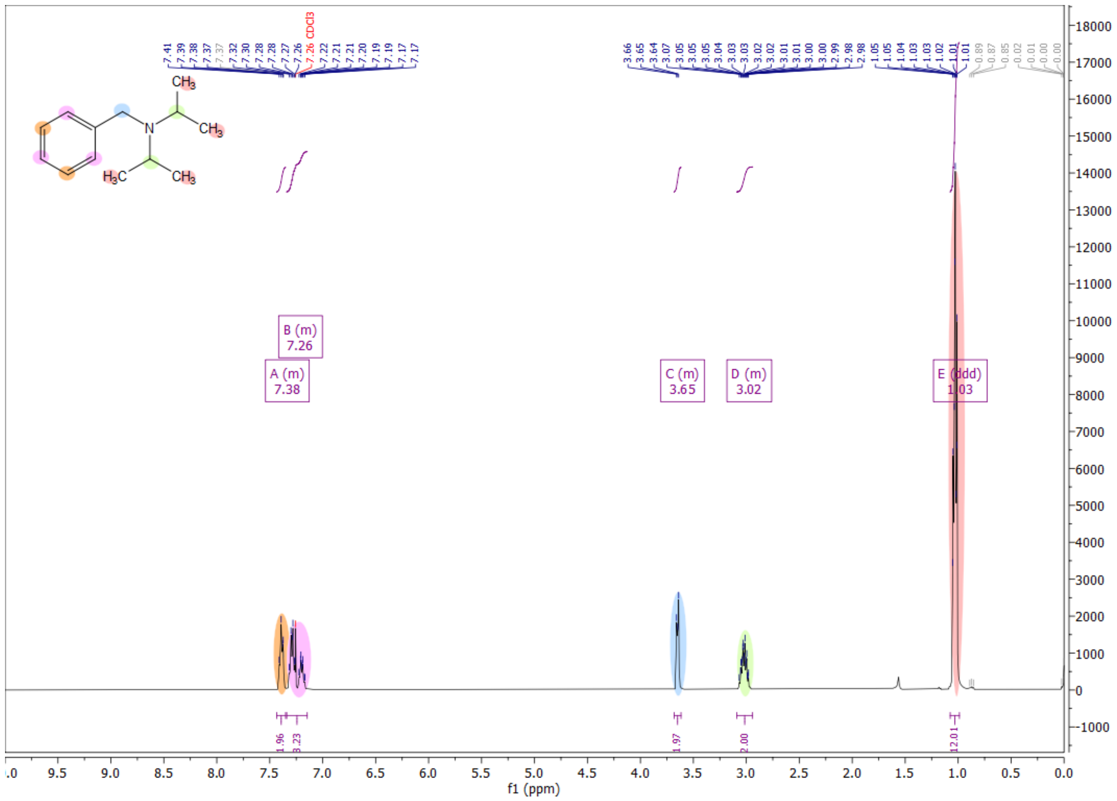

Figure S33: ^{1}H-NMR of Benzyldiisopropylamine.

3. References

S1 M. V. Jiménez, J. Fernández-Tornos, M. González-Lainez, B. Sánchez-Page, F. J. Modrego, L. A. Oro and J. J. Pérez-Torrente, Mechanistic studies on the N -alkylation of amines with alcohols catalysed by iridium(i) complexes with functionalised N-heterocyclic carbene ligands, Catal. Sci. Technol., 2018, 8, 2381–2393.

S2 D. Yan, X. Wu, J. Xiao, Z. Zhu, X. Xu, X. Bao, Y. Yao, Q. Shen and M. Xue, n -Butyllithium catalyzed hydroboration of imines and alkynes, Org. Chem. Front., 2019, 6, 648–653.

S3 X. Zhu, Q. Zhang and W. Su, Solvent-free N-arylation of amines with arylboronic acids under ball milling conditions, RSC Adv., 2014, 4, 22775.

S4 F. Mao, D. Sui, Z. Qi, H. Fan, R. Chen and J. Huang, Heterogeneous cobalt catalysts for reductive amination with H 2 : general synthesis of secondary and tertiary amines, RSC Adv., 2016, 6, 94068–94073.

S5 H. Kato, I. Shibata, Y. Yasaka, S. Tsunoi, M. Yasuda and A. Baba, The reductive amination of aldehydes and ketones by catalytic use of dibutylchlorotin hydride complex, Chemical communications (Cambridge, England), 2006, 4189–4191.

S6 S. Dong, Z. Zong, N. Sun, B. Hu, Z. Shen, X. Hu and L. Jin, Hydrosilylative reduction of secondary amides to amines catalyzed by geometry-constrained NNN -cobalt complexes, New J. Chem., 2023, 47, 5603–5610.

S7 J. Wu, S. Lu, D. Ge and H. Gu, Highly efficient and eco-friendly synthesis of tertiary amines by reductive alkylation of aldehydes with secondary amines over a Pt nanowires catalyst, RSC Adv., 2015, 5, 81395–81398.

S8 L. Blackburn and R. J. Taylor, In situ oxidation-imine formation-reduction routes from alcohols to amines, Organic letters, 2001, 3, 1637–1639.
